# Variable Splitting and Fusing for Image Phase Retrieval

**DOI:** 10.3390/jimaging10100249

**Published:** 2024-10-12

**Authors:** Petros Nyfantis, Pablo Ruiz Mataran, Hector Nistazakis, George Tombras, Aggelos K. Katsaggelos

**Affiliations:** 1 Department of Physics, National and Kapodistrian University of Athens, 15784 Athens, Greece; 2 Chartboost, 08018 Barcelona, Spain; 3 Department of Electrical Engineering and Computer Science, Northwestern University, Evanston, IL 60208, USA

**Keywords:** phase retrieval, alternating optimization, non-convex optimization, microscopy, inverse image problems

## Abstract

Phase Retrieval is defined as the recovery of a signal when only the intensity of its Fourier Transform is known. It is a non-linear and non-convex optimization problem with a multitude of applications including X-ray crystallography, microscopy and blind deconvolution. In this study, we address the problem of Phase Retrieval from the perspective of variable splitting and alternating minimization for real signals and seek to develop algorithms with improved convergence properties. An exploration of the underlying geometric relations led to the conceptualization of an algorithmic step aiming to refine the estimate at each iteration via recombination of the separated variables. Following this, a theoretical analysis to study the convergence properties of the proposed method and justify the inclusion of the recombination step was developed. Our experiments showed that the proposed method converges substantially faster compared to other state-of-the-art analytical methods while demonstrating equivalent or superior performance in terms of quality of reconstruction and ability to converge under various setups.

## 1. Introduction

Phase Retrieval (PhR) is the inverse problem of reconstructing a signal when only the magnitude of its Fourier transform is available. A typical example is Ptychography, where a set of interference patterns is generated by illuminating a specimen at various centering positions, at preset intervals between them.

The obtained measurements contain only magnitude information and computational imaging methods can produce an image of the specimen by combining the information of each pattern at different positions. Examples of phase retrieval applications include microscopy [1], X-ray crystallography [2,3,4], coherent diffraction imaging [5], array imaging [6], blind deconvolution [7], acoustics [8], interferometry [9] and astronomy [10]. The problem can be stated more generally as retrieving x from measurements of the type

(1) $$y_{i}={\left|h_{i}^{*}x\right|}^{2}+\eta_{i},\;\;\;i=1,\ldots ,M,$$

where $h_{i}^{*}$ is the *i*-th sampling vector and $\eta_{i}$ is an additive noise term. Phase Retrieval belongs to the class of non-linear non-convex optimization problems. Theoretical advances in random linear operators enabled the application of the solution uniqueness principles of random underdetermined linear systems (Compressive Sensing) to the quadratic measurements problem of Phase Retrieval.

In detail, the original signal x can be recovered, when the sampling vectors $h_{i}^{*}$ are drawn randomly, following a probability distribution, such as a Gaussian [11]. In practice, this can be achieved by masking the sample with binary masks or optical grating [12]. Additionally, in the general case the uniqueness of the solution cannot be guaranteed unless a sufficient number of samples exceeding the signal size is available.

### 1.1. Related Work

The most widely accepted reconstruction methods before the invention of modern techniques were the Gerchberg–Saxton [13] and Fienup [10] algorithms. Both methods are based on iterative nonlinear projections for the refinement of their estimates. There was no guarantee for the convergence of these algorithms and solutions could typically be obtained under special conditions for the input signals or special initializations relying on prior information. Subsequent research produced nonconvex iterative methods such as the extended Ptychographic Iterative Engine [14] and Difference Map [15].

The advent of Compressive Sensing [16] and its theoretical connection to the problem of Matrix Completion [17] led to the development of new theoretical results on Phase Retrieval. Specifically, in [11,12], Candès et al. approached the problem via the “Lifting” technique, where a convex relaxation allows to search for the solution to the problem in the space of positive semidefinite matrices, through trace norm minimization with the problem being recast as one of matrix completion. This formulation, in conjunction with special properties for the transform operators, namely having a randomness property, allowed to provide guarantees for convergence to a unique solution for the problem, given the availability of sufficient samples [8,18,19,20].

The matrix completion formulation of Phase Retrieval and related semidefinite programming methods [21] are computationally prohibitive for any sizeable signal, for example, a high-resolution image, since they involve the manipulation of very large dimensional variables. In response to this, a number of efficient non-convex optimization methods based on Stochastic Gradient Descent (SGD) were developed, namely Wirtinger Flow and Amplitude flow [19,22,23], where random operator properties and a good initial estimation of the solution are used in order to guarantee their convergence. The success of SGD methods led to the development of numerous modifications, aiming to improve their convergence and noise resilience performance, see [24,25,26,27,28] among others.

Beyond Wirtiger flow-related studies, research on Phase Retrieval produced algorithms based on non-linear optimization [29], alternating minimization [30,31] and ADMM methods [32]. Furthermore, the problem was addressed from the perspective of Basis Pursuit convex optimization [33,34], low rank matrix completion [35,36] and Total Variation minimization [37]. Ref. [38] explored insights on the geometry of the problem of Phase Retrieval. In [39] the Generalized Approximate Message Passing framework was applied to Phase Retrieval.

The ongoing developments in Deep Learning have resulted in a plethora of Neural Network methods for Phase Retrieval. Deep Learning methods include direct network approaches, in which Neural Networks are trained with specific datasets in order to learn the function mapping input to output [40,41]. Beyond dataset-based approaches, a number of physics-oriented methods were developed. Physics-based methods utilize a model of the dynamics of the sensing system as a prior driving the training or inference process of Neural Networks. In such methods, the Neural Network can act as a regularizer in an iterative estimation process, confining the estimates to certain spaces [42,43,44]. Alternatively, the iterations of an analytical process for Phase Retrieval can be mapped on the layers of a Neural Network [45,46].

Neural Networks have also been combined with numerical methods in order to refine imperfect estimates into high-quality reconstuctions [47,48]. Untrained neural network physics-based methods have been used to alleviate various problems that stem from the lack of adequate or good-quality training data as well as imperfect modeling of the signal propagation [49,50,51]. Deep learning has also been utilized to optimize the design of coded diffraction patterns [52].

Alternating optimization algorithms have also utilized Neural Networks as regularizers [53,54] in order to improve noise stability and achieve better performance. The ADMM has also been used as a physical model for untrained network methods [55,56]. An overview of Deep Learning techniques for Phase Retrieval related to a wide variety of applications and sensing configurations is provided in [57].

### 1.2. Our Contribution

We introduce a solver for the non-convex optimization problem of Equation (1), concerning real signals, which belongs to the category of alternating minimization algorithms. Our method differs from traditional alternating estimation algorithms since it does not involve the type of updates used in the Gerchberg–Saxton and Fienup methods, where successive nonlinear projections to desirable sets are used to refine the solution. Our study follows the line of theoretical results on Phase Retrieval with random sensing operators, specifically the interpretation of Phase Retrieval as a Matrix Completion problem and uses established results on the uniqueness of the solution in the space of rank-1 Hermitian matrices [11,22] in order to derive a nonconvex split variables formulation (see Equation (3)).

Our formulation differs from other alternating minimization methods such as [30,31], which estimate a phase and a solution vector. Instead, it reformulates the optimization problem by using two vectors for the estimated solution, expanding the search space to all rank-1 matrices (Equation (4)). The paper [32] shares the same optimization problem formulation as the one presented here (Equation (7)). However, the main theoretical and algorithmic innovation of our study lies in that it unveils and utilizes an implicit geometric relation of the split variables at optimal points in order to enforce their equality, by calculating a recombination of them at each iteration (Equation (13)) effectively restricting the estimated solution space to rank-1 Hermitian matrices. This, is without  the need for additional regularization terms in the objective function, such as the ones used in various versions of the Alternating Direction Method of Multipliers.

As a result, the proposed updated equations correspond to a fundamentally different formulation of the Alternating Direction Method compared to the one presented in [32] (also see Section S5 in Supplementary File). To the best of our knowledge, the updated equations of the proposed method are not equivalent to any existing iterative method for Phase Retrieval. Empirical results show that the inclusion of the recombination step is necessary for the algorithm to converge (see Section S6 of the Supplementary File). Furthermore, since we are not aware of any results that correspond to the recombination step in the literature, we provide a theoretical analysis exploring the convergence properties of the proposed non-linear optimization method, in order to justify its general applicability.

An experimental comparison shows that our method demonstrates superior convergence properties compared to state-of-the-art analytical methods, in terms of its ability to converge for various numbers of available samples, processing time and accuracy under the presence of noise.

### 1.3. Paper Structure

The rest of the paper is organized as follows: In Section 2, the problem formulation and an introduction of the proposed optimization method is provided along with a theoretical analysis of its convergence. Section 3 contains experimental results, where the proposed method is evaluated and compared against other analytical Phase Retrieval solvers.

## 2. Method

### 2.1. Problem Formulation

In our experiments and analysis, we consider the case of observations, which are acquired according to the following forward model

(2) $$y_{ij}={\left|f_{i}^{T}W_{j}x\right|}^{2}+\eta_{ij},\;\;\;i=1,\ldots ,N\;\;\mathrm{and}\;\;j=1,\ldots ,K,$$

that is a matrix $Y\in R^{N\times K}$ with entries $y_{ij}$, observed by applying a set of *K* masks $W_{j}\in C^{N\times N}$ (diagonal matrices) to the original signal $x\in R^{N\times 1}$ and measuring the squared magnitude ${\left|.\right|}^{2}$ of each element of the Discrete Fourier Transform (DFT) of the masked signal, where the DFT matrix is represented by $F\in C^{N\times N}$ with rows $f_{i}^{T},i=1,\ldots ,N$. Finally, a noise term $\eta_{ij}$ is added to the observation.

This observation model corresponds to a sample acquisition system where the sampled image is first modulated before it is acquired by the sensor. Examples of such systems are masking [58] and ptychography [59]. In this study the mask elements are sampled randomly from the set {1,−1,i,−i}, where *i* represents the imaginary unit. A modulation of this kind could be achieved with a phase-shift mask, without precluding the use of other coded patterns achievable with a modulated illumination beam or ptychography.

According to [12], the Phase Retrieval problem can be transformed into the following matrix completion problem

(3) $$\begin{array}{cc} \; & \mathrm{find}\;\;X\;\;\mathrm{subject}\;\mathrm{to}\;\\ \; & \left(i\right)\;\mathrm{rank}(X)=1,\;\;\left(\mathrm{ii}\right)\;X⪰0\;\;(\mathrm{positive}\;\mathrm{semidefinite}\;\mathrm{matrix}),\\ \; & \left(\mathrm{iii}\right)\;{\left(y_{ij}-<H_{ij},X>\right)}^{2}\leq \eta_{ij},i=1,\ldots ,N,j=1,\ldots ,K, \end{array}$$

where X is a N×N matrix, $H_{ij}=Re\left\{W_{j}^{*}f_{i}f_{i}^{T}W_{j}\right\}$ and the operator <.,.> is the Frobenius inner product. According to the same work [12], given random sampling vectors and an adequate number of samples, the optimal matrix X is unique and can be used to identify the vector x up to a global phase. Since it holds that $X={\mathrm{xx}}^{T}$ at the global minimum, the search space for the matrix X is the positive semidefinite cone.

Against this backdrop, we use a split variable formulation to solve the problem in Equation (3), and to enforce the rank-1 constraint in Equation (3) (i), by factorizing the matrix X as

(4) $$X=ba^{T},$$

where a and b are vectors in $R^{N}$. This formulation allows the observation constraints, in Equation (3) (iii) to be written as

(5) $${\left(<H_{ij},X>-y_{ij}\right)}^{2}={\left(a^{T}H_{ij}b-y_{ij}\right)}^{2},$$

since

(6) $$<H_{ij},X>=Tr\left(H_{ij}X\right)=Tr\left(H_{ij}ba^{T}\right)=Tr\left(a^{T}H_{ij}b\right)=a^{T}H_{ij}b.$$

Splitting the variable x into a and b is the reason why the real part in the definition of the sampling operator $H_{ij}=Re\left\{W_{j}^{*}f_{i}f_{i}^{T}W_{j}\right\}$ is used. For a sampling vector $h\in C^{N}$, and denoting $h_{r}$ and $h_{i}$ its real and imaginary parts, respectively, $|h^{T}{x|}^{2}=\left(h^{T}x\right)\left({\bar{h}}^{T}\bar{x}\right)$ = ${\left(h_{r}^{T}x\right)}^{2}+{\left(h_{i}^{T}x\right)}^{2}$. For the split variables $a,b\in R^{N}$$\left(h^{T}a\right)\left({\bar{h}}^{T}\bar{b}\right)$ = $h_{r}^{T}ah_{r}^{T}b$ + $h_{i}^{T}ah_{i}^{T}b$ + i$h_{i}^{T}ah_{r}^{T}b$ − i$h_{r}^{T}ah_{i}^{T}b$. When a≠b the imaginary terms do not cancel out and since the value must be real, only the real part of the sampling vector times its transpose is retained.

Similar approaches can be found in the literature on the matrix completion problem. For instance, in [60], matrix X is factorized as $X=ab^{T}$, where a and b are matrices of size N×r and its nuclear norm is minimized through alternating estimation of the values of a and b. The main difference between this approach and our method is that in the case of PhR, we can enforce the rank-1 constraint and equality of a and b.

Thus, the PhR problem can be interpreted as one of matrix completion with split variables. Solving for a and b does not necessarily lead to positive-semidefinite (PSD) solutions for X, as is required in Equation (3) (ii). To avoid this problem, we enforce a stronger constraint, a=b, which implies that the only non-zero eigenvalue of X, $\lambda_{1}\left(X\right)=a^{T}a$, is positive, and therefore, X⪰0.

Finally, the PhR problem can be formulated as the following optimization problem

(7) $$\underset{a,b}{arg min}\sum_{ij}{\left(a^{T}H_{ij}b-y_{ij}\right)}^{2},\;s.t.\;a=b.$$

### 2.2. Proposed Optimization Algorithm

The optimization problem of Equation (7) can be recast as

(8) $$\sum_{ij}{\left(a^{T}H_{ij}b-y_{ij}\right)}^{2}={∥H_{a}b-y∥}^{2}$$

where ∥.∥ denotes the Frobenius norm of a vector and $H^{a}$ is a KN×N matrix whose rows are defined as

(9) $$H_{a}\left(i+N\left(j-1\right),:\right)=a^{T}H_{ij},\;i=1,\ldots ,N,j=1,\ldots ,K$$

and y denoting the vector obtained by stacking the columns of matrix Y.

We propose a method that alternates between the estimations of a and b. At iteration *n*, the vectors a and b are calculated by solving

(10) $$\begin{array}{cc} \; & a^{n}=\underset{a}{arg min}{∥H_{b}a-y∥}^{2} \end{array}$$

(11) $$\begin{array}{cc} \; & b^{n}=\underset{b}{arg min}{∥H_{a}b-y∥}^{2} \end{array}$$

Alternatingly solving Equations (10) and (11) does not result in convergence. An empirical investigation (please see Section S6 in the Supplementary File) reveals the geometric properties of the local minima. It can be seen that the vectors a and b tend to attain resting positions that lie on opposing sides of the solution vector.

Consequently, to enforce a=b, we introduce a refinement step in the process which replaces the vectors a and b with their mean

(12) $$a^{n}=b^{n}=\frac{1}{2}\left(a^{n}+b^{n}\right).$$

The convergence analysis of the algorithm presented in Section 2.3 shows that the second estimation step can be skipped, as calculating the local minimum of only one of the variables suffices for the recombination step to provide an estimate that is closer to the solution.

The recombination step then becomes

(13) $$a^{n}=b^{n}=\frac{1}{2}\left(a^{n}+b^{n-1}\right).$$

The final form of the proposed optimization method is summarized in Algorithm 1.
**Algorithm 1** Proposed Algorithm for Phase RetrievalGiven input $y\in R^{M}$, *K* known masks $W_{k}\in C^{N\times N}$ and tolerance ϵInitialize $\left\{a^{0}=b^{0}=Initialize\left(Y,W\right)\right\}$n=0**while** $|a^{n}-a^{\left(n-1\right)}|\;\geq \epsilon$ **do**   n=n+1   $b^{n}={\left({\left(H_{a^{\left(n-1\right)}}\right)}^{T}\left(H_{a^{\left(n-1\right)}}\right)\right)}^{-1}{\left(H_{a^{\left(n-1\right)}}\right)}^{T}y$   $b^{n}=a^{n}=0.5\left(a^{n-1}+b^{n}\right)$**end while**

The estimation of $b^{n}$ involves the application of a conjugate gradient solver. If *p* the number of maximum allowed iteration of the Conjugate Gradient solver [61] and *q* the maximum allowed iterations of Algorithm 1, the total computational complexity is (see Section S3 in the Supplementary Material) O(q(3p+1)KNlogN+q(4p+2)KN+10qpN) basic operations.

### 2.3. Convergence Analysis

In this subsection, theoretical results on the convergence of Algorithm 1 are presented. This analysis is related to real signal and variable vectors $x,a,b\in R^{N}$.

#### 2.3.1. Equations at Local Minima

Let d and e be the error vectors, such as

(14) a=x+dandb=x+e.

At each iteration, the vector a is computed via a Linear Least Squares system solution

(15) $$a={\left(H_{b}^{T}H_{b}\right)}^{-1}H_{b}^{T}y.$$

At this optimal point, it holds that

(16) $$\begin{array}{cc} \; & H_{b}^{T}H_{b}a=H_{b}^{T}y=H_{b}^{T}H_{x}x. \end{array}$$

By using the definition of the error vectors of Equation (14), multiplying the left sides of Equation (16) with the transpose of the vector g=e+d and after some algebra (please see Section S1 of the Supplementary File), we obtain

(17) $$g^{T}H_{b}^{T}H_{b}g=g^{T}H_{b}^{T}H_{e}e.$$

The algorithm will converge, if at each iteration it holds that

(18) $$\frac{1}{2}∥g∥\leq min\left(∥d∥,∥e∥\right).$$

Assuming that only b (or equivalently a) is optimized, the value of d is left unchanged. Thus, if at each iteration

(19) $$\frac{1}{2}∥g∥\leq ∥e∥,$$

the algorithm will converge.

#### 2.3.2. Norm of g

We proceed to examine the relation of the norms of the vectors g and e. From the triangle inequality and Equation (17) follows that

(20) $$|E\left\{g^{T}H_{b}^{T}H_{b}g\right\}-E\left\{g^{T}H_{b}^{T}H_{e}e\right\}|\;\leq \;|E\left\{g^{T}H_{b}^{T}H_{b}g\right\}-g^{T}H_{b}^{T}H_{b}g|\;+\;|g^{T}H_{b}^{T}H_{e}e-E\left\{g^{T}H_{b}^{T}H_{e}e\right\}|.$$

We establish close-form solutions for the expectation and concentration inequality for the quantities $g^{T}H_{b}^{T}H_{b}g$ and $g^{T}H_{b}^{T}H_{e}e$ (please see Section S2 of Supplementary Material).

Combining inequality (20) with Equations (S41), (S43) and inequalities (S93), (S108), leads to

(21) $$\begin{array}{c} {|∥b∥}^{2}{∥g∥}^{2}\left(0.5+1.5cos^{2}\theta_{b}^{g}\right)-{(∥e∥}^{2}g^{T}b+e^{T}ge^{T}b)|\leq {(\delta ∥g∥}^{2}+\delta ∥g\;|∥e∥|cos\theta_{e}^{g}\left|\right), \end{array}$$

which holds with very high probability.

Since $cos\theta_{e}^{g}\in \left[-1,1\right]$, in the limit cases inequality (21) after some algebra becomes

(22) $$∥g∥=\frac{∥e∥}{\left(∥b∥\mp \frac{\delta }{∥b∥}\right)}\left(\frac{cos\theta_{g}^{b}+cos\theta_{g}^{e}cos\theta_{e}^{b}}{0.5+1.5cos^{2}\theta_{g}^{b}}\pm \delta \frac{1}{∥e∥∥b∥}\right)∥e∥.$$

#### 2.3.3. Convergence

Since ∥g∥ ≥0, from inequality (19) and Equation (22) the algorithm will converge if at each iteration

(23) $$0\leq \frac{∥e∥}{\left(∥b∥\mp \frac{\delta }{∥b∥}\right)}\left(\frac{cos\theta_{g}^{b}+cos\theta_{g}^{e}cos\theta_{e}^{b}}{0.5+1.5cos^{2}\theta_{g}^{b}}\pm \delta \frac{1}{∥e∥∥b∥}\right)\leq 2$$

To declutter the equations, we define

(24) $$\frac{∥e∥}{∥b∥\mp \frac{\delta }{∥b∥}}=\xi^{-1},cos\theta_{g}^{e}cos\theta_{e}^{b}=\zeta ,cos\theta_{g}^{b}=u\;\mathrm{and}\;\lambda =\delta \frac{1}{∥e∥∥b∥}$$

for which it holds that u,ζ∈[−1,1], λ≥0 and ξ≥0.

To find the conditions that satisfy inequalities (23), we rewrite it as

(25) $$0\leq \frac{u+\zeta }{1+3u^{2}}\pm \frac{\lambda }{2}\leq \xi .$$

Since ζ is upper bound by 1,

(26) $$0\leq \frac{u+\zeta }{1+3u^{2}}\pm \frac{\lambda }{2}\leq \frac{u+1}{1+3u^{2}}\pm \frac{\lambda }{2}\leq \xi .$$

A larger value for ξ corresponds to a smaller distance tolerance for the error vector e.

Assuming a pessimistic scenario and minimum value of such a distance, the bound of ξ is

(27) $$\frac{u+1}{1+3u^{2}}\pm \frac{\lambda }{2}\leq \xi .$$

**Figure 1:**
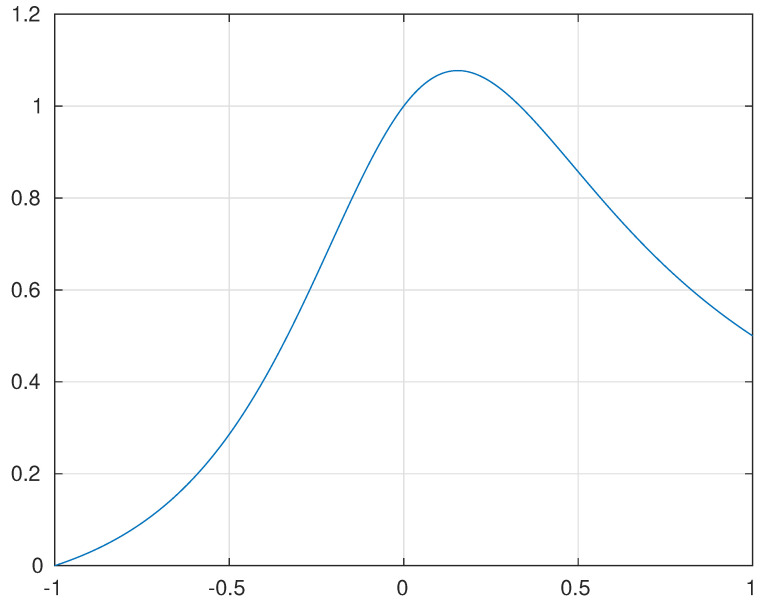
Plot of the function $f\left(u\right)=\frac{u+1}{3u^{2}+1}$ against the variable $u=cos\theta_{g}^{b}$.

An upper bound for $\frac{u+1}{3u^{2}+1}\leq 1.08$ is numerically computed (see Figure 1), thus in the worst case

(28) $$0\leq \xi^{-1}\leq \frac{1}{1.08\pm 0.5\lambda }.$$

Reinstating the original variables

(29) $$0\leq \frac{∥e∥}{∥b∥\mp \frac{\delta }{∥b∥}}\leq \frac{1}{1.08\pm \frac{\delta }{2∥e∥∥b∥}}.$$

From the non-negativity constraint, the denominators must be positive.

After some algebra, the geometric constraint becomes

(30) $$\frac{∥e∥}{∥b∥}\leq \frac{1}{1.08}-\frac{1.5\delta }{{∥b∥}^{2}}$$

or

(31) $$∥e∥∥b∥\leq \frac{1}{1.08}{∥b∥}^{2}-1.5\delta ,$$

where since the right term of the inequality is an upper bound, only the term with the minus sign was retained.

Solving the quadratic equation and after some algebra, the condition of convergence becomes

(32) $$\mu =\frac{∥e∥}{∥b∥}\leq \frac{1}{1.08}-\frac{1.08}{2}\sqrt{\frac{6\delta }{1.08}}.$$

It remains to express the constraint as a relation between the norm of the error and solution vectors.

From the definition of the error vector we have

(33) $$b=x+e\Leftrightarrow {∥b∥}^{2}={∥x∥}^{2}+{∥e∥}^{2}+2x^{T}e.$$

The minimal value for ∥e∥ is attained, when $cos\theta_{e}^{x}=-1$, i.e., when b, e and x are collinear.

At this geometric point, it holds that

(34) ∥e∥+∥b∥ = ∥x∥

or

(35) $$∥e∥(1+\frac{1}{\mu })=\;∥x∥.$$

From inequality (32) it follows that

(36) $$\mu \leq \frac{1}{1.08}-\frac{1.08}{2}\sqrt{\frac{6\delta }{1.08}}<1$$

or

(37) $$\frac{1}{\mu }\geq \frac{1.08}{1-\frac{1.08^{2}}{2}\sqrt{\frac{6\delta }{1.08}}}\geq 1.08.$$

Combining Equation (35) and inequality (37),

(38) $$∥x∥\;=\;∥e∥(1+\frac{1}{\mu })\geq \;∥e∥2.08.$$

From inequality (38) it follows that an initialization which results in a value ∥e∥ ≤0.48∥x∥ will always lead to convergence.

The above result assumes the worst case scenario for the value of the variable ζ, as well as the geometric relations of the vectors e, b and x.

Assuming that the uncertainty term relying on δ is equal to zero for ease of exposition, the best possible scenario is the case where ζ=−1 for which μ∈[0,∞], or stated otherwise, any initial value of ∥e∥ results to a smaller error norm.

In a case where ζ≈0, which implies that the vectors e, b and g are not aligned, a value for the error vector where ∥e∥ ≤3.5∥b∥, will also lead to a lower error.

The convergence analysis presented in this section relies mainly on the terms of the right side of inequality (17) attaining small values or equivalently that for various instantiations of the masks, the quantities $g^{T}H_{b}^{T}H_{b}g$ and $g^{T}H_{b}^{T}H_{e}e$ are highly concentrated around their expected value. The type of random masks examined endows the forward operator with special statistical properties that promote the high concentration. The convergence analysis presented in this study assumes that for each mask element, it is true that

(39) E{w}=0,

(40) $$E\{w^{2}\}=0,$$

(41) $${E\{|w|}^{4}{\}\;=2E\{|w|}^{2}\}$$

and

(42) $$E\{|w{|}^{2}\}=1.$$

Masks with elements that belong to {1,−1} or {i,−i} will also lead to convergence. Empirical tests show that for the proposed algorithm to function well the zero expectation property must be maintained. Beyond the aforementioned, other families of masks that enhance the concentration of measure of the sensing matrices, such as Designed Coded Diffraction [62], can be utilized.

## 3. Results

This section contains experiments showing the performance of the proposed algorithm in terms of numerical error and execution time. The proposed method is compared with other analytical methods such as the Wirtinger Flow Phase Retrieval (WF) [63], Truncated Wirtinger Flow (TWF) [23] Truncated Amplitude Flow (TAF) [19], the Momentum median reweighted Truncated Amplitude Flow (MRTAF) [28] and the PhaseSplit [32] method which shares the same formulation as the method proposed in this study.

Notice that the forward model of TAF, or MRTAF uses the non-squared magnitudes as input, but the proposed method, WF, TWF and PhaseSplit consider the squared magnitudes. Therefore, the noisy measurements of one category of algorithms do not result in the same SNR for the other and cannot be used directly. However, we compare the methods by adding a level of noise which results in the same SNR to the magnitudes and squared magnitudes observations, respectively.

Each method has parameters that control its performance. The standard parameters were used, as provided by their authors.

In the experiments three initialization methods were considered, one based on the Truncated Wirtinger Flow spectral initialization (TWF) introduced in [23], one based on the Truncated Amplitude Flow spectral initialization (TAF) introduced in [19] and a proposed positive random numbers initialization method (see Section S4 of the Supplementary File).

The proposed method was implemented in MATLAB © and MATLAB © implementations of WF, TAF and TWF were downloaded from the respective websites, see Table 1. All test images shown were obtained by the USC-SIPI Image Database (The USC-SIPI test image dataset can be found in https://sipi.usc.edu/database/ (accessed on 20 July 2024)). The implementation of PhaseSplit and MRTAF where not readily available and were implemented by us since they correspond to minor modifications of the proposed and TAF methods, respectively.

In the simulations, three different images were used. “Lena” of sizes 64×64 and 128×128, “Cameraman” of size 256×256, and “Man” of sizes 512×512 and 1024×1024.

In the experiments, the proposed algorithm stops executing when the distance between the last two estimated output values becomes lower than a given threshold, set to $10^{-15}$.

To evaluate the performance of the proposed method we use the following metric, which is the square root of the metric proposed in [11]

(43) $$err=\frac{min_{\phi \in [0,2\pi ]}∥e^{-i\phi }\hat{x}-x∥}{∥x∥}$$

This metric takes into account the fact that the original signal x and any other signal obtained by a global phase delay of x always produces the same observation.

For the case where x is real, this is

(44) $$err=\frac{min∥\hat{x}\pm x∥}{∥x∥}$$

We generate simulated observations according to the acquisition model introduced in Equation (2).

We consider a different number of masks in our simulations, that is K=2,4,8,16.

The elements of each mask $W_{j}$, j=1,…,K, are uniformly drawn from the Coded Diffraction Patterns dictionary {1,−1,i,−i} (see [22]). These diffraction patterns correspond to physically realizable acquisition systems, where only a phase delay is introduced using appropriate masking.

Different levels of AWGN noise were considered in our simulations, with SNRs equal to ∞, 30, 24, 20 and 10 dB.

### 3.1. Initialization Quality

The performance of the initialization method proposed in Section S4 of the Supplementary File was evaluated first.

Table 2 shows the normalized error obtained by the proposed random initialization method, TWF initialization method and TAF initialization method, for the noiseless case with K=2,4,8,16.

The proposed random initialization method produces estimates of similar quality for all cases. The error of the proposed initialization method increases with the size of the input image but is similar for different numbers of masks with each size.

We observe that TWF and TAF need at least *K* = 8 and *K* = 4 masks, respectively, to obtain a normalized error smaller than one, but the proposed initialization method can obtain normalized errors smaller than one, even with *K* = 2.

Table 3 shows the normalized error obtained by the proposed initialization method, TAF initialization method and TWF initialization method, for SNR = 20 dB and K=2,4,8,16. The proposed method performs similarly to the noiseless case.

The TWF method needs more than *K* = 8 masks to obtain a normalized error less than 1 while the TAF initialization begins to do so with *K* = 4 masks. The quality of the estimates of the proposed initialization method does not change substantially by the presence of noise compared to the noiseless case.

Table 4 shows the time required for the initialization routine to return for the WF, TWF and TAF methods. The Proposed method is omitted since it has effectively zero execution time (for example, it is 0.01 s for K=16 and 1024×1024 images, the slowest case in the experiments conducted in this work). The results are for various image sizes and K=2,4,8,16 in the noise-free observations case.

The noisy cases are not shown but would have the same return times since the presence of noise does not affect the execution times of the iterations, and the iterations number is predefined. We observe that the times required grow with the number of masks and image sizes, which is expected due to the higher computational complexity. The WF method is the fastest, with the second fastest being the TWF and TAF the slowest.

This pattern reflects the higher complexity of the truncation calculations in each iteration. Generally, the WF, TWF and TAF initializations can consume substantial computational resources, with execution times equivalent to 40% of the reconstruction time in the cases where noise is present.

### 3.2. Reconstructions with Noise-Free Observations

**Figure 2:**
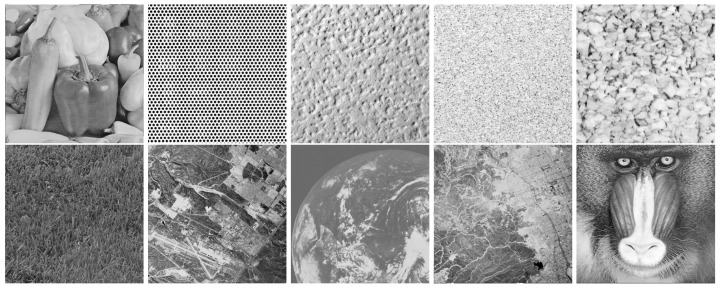
Reconstructions of 512×512 images for *K* = 2 using the proposed method.

Figure 2 shows the reconstructions of 10 test images for *K* = 2 masks in the noiseless case, using the proposed method. In all cases the images were perfectly reconstructed.

In all noise-free cases, the proposed method is able to recover the original signal. Regarding the compared methods, TWF, TAF, and MRTAF also recover the exact solution in all cases. However, WF only recovers the exact solution when K>4. For K=4, WF converges to an inexact reconstruction of the image.

**Figure 3:**
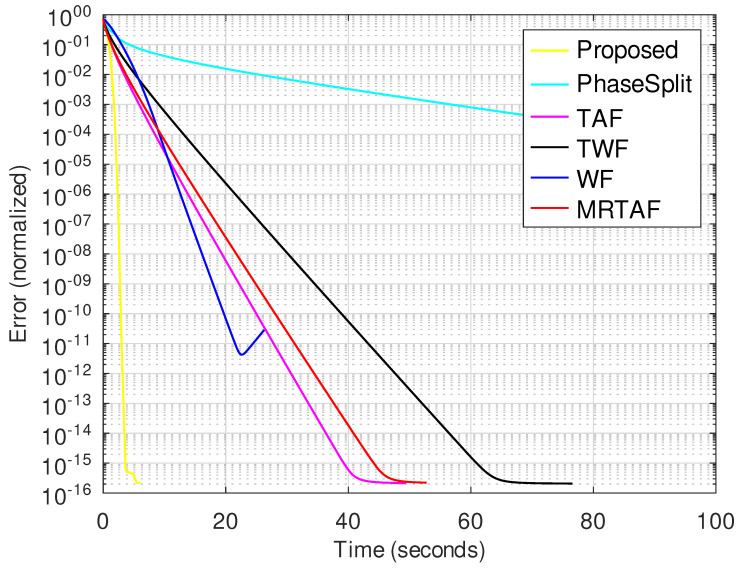
Normalized error vs. time for image size 256×256 with *K* = 8 in the noiseless case.

Figure 3 shows the evolution of the reconstruction error with time for all methods tested. The proposed method converges to a solution substantially faster than the compared methods. Beyond this WF, TAF and MRTAF are the methods that converge relatively faster. The PhaseSplit method converges more slowly than the proposed and SGD-based methods and its convergence behavior is very sensitive to changes in its parameters (also see Section S5 of Supplementary Material).

Table 5 shows the execution time taken for all methods to achieve exact reconstruction for various input sizes and K=4,8 for both the proposed and TAF initializations, with the latter being considered the best available spectral initializer. In some of the experiments, the WF method failed to reach an exact solution and terminated early with a solution of normalized error typically close to 0.03.

In all experiments, the proposed method is faster than all the compared methods. More specifically, we observe that the proposed method is approximately four times faster than the second WF fastest method.

In the next experiment, the convergence success rate is measured. We generate 100 random signals of size 32×32 and apply the observation model in Equation (3) to generate 100 noise-free observations. Then, we apply the compared methods and calculate the percentage of signals that have been successfully recovered.

The experiment is repeated for two different ways of generating the signal. More specifically, we use a uniform distribution on the interval [0, 1], and a standard Gaussian distribution with mean 0 and covariance the identity matrix. The results of this experiment are shown in Table 6.

For signals generated with the Gaussian distribution, the proposed method converges with higher probability when the TAF initialization is used, since the quality of the proposed initialization is worse, as was seen in the previous experiment.

The failures in convergence were associated with poor initialization quality up to K=6. Beyond this threshold there is enough information for the spectral initialization to be of good quality. More than six masks also suffice for the method to converge, regardless of the initialization, which can be deduced by the success rate for a general real signal when the initialization only contains positive numbers. When the TAF initialization was used, all methods had progressively better success rates with higher *K*.

The WF method follows the same converge patterns, regardless of initialization and signal type with only the number of masks determining the rate of success. TAF and TWF always failed when the proposed random initialization was used for general real signals due to the poor quality of the initialization.

The proposed method and PhaseSplit had higher success rates for lower *K* compared to all other methods.

Generally, the proposed method produces reconstructions for the noise-free case comparable to the obtained ones by the state-of-the-art methods, with the advantage of being much faster.

The proposed random initialization method also leads to acceptable initializations in practice.

The proposed method works when the lower theoretical bound of *K* = 2 masks is available, given that a good-quality initialization is available. Reconstructions with *K* = 2 masks have only been reported in the TAF paper [19], for real valued sampling vectors. PhaseSplit could also produce a similar level of performance to the proposed method when its parameters were finely tuned.

The proposed method also converges with *K* = 2 masks in the case of complex sampling vectors.

### 3.3. Reconstructions with Noisy Observations

**Figure 4:**
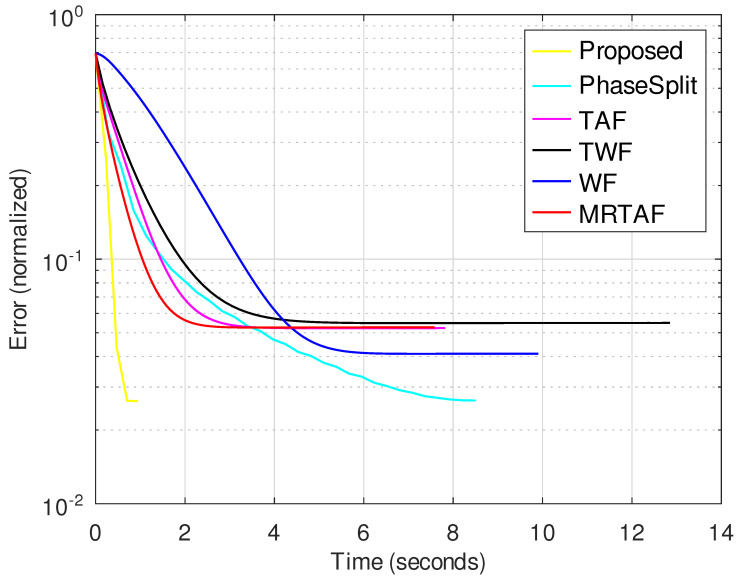
Normalized error vs. time for image size 256×256 with SNR = 24 dB and *K* = 8.

**Figure 5:**
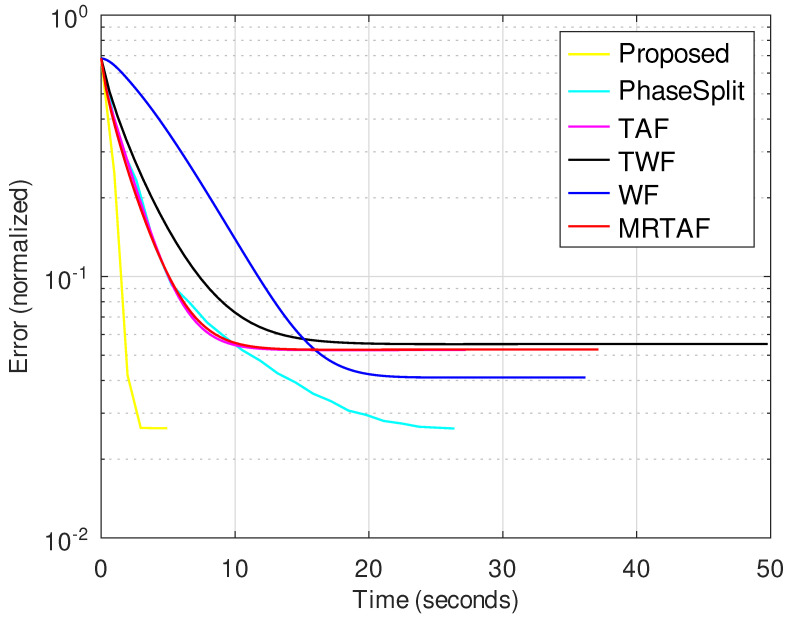
Normalized error vs. time for image size 512×512 with SNR = 24 dB and *K* = 8.

In this subsection, we evaluate the performance of the compared methods for noisy observations. Figure 4 and Figure 5 show the evolution of the normalized error for all compared methods for SNR = 24 dB and *K* = 8, when the image size is 256×256 and 512×512, respectively.

Table 7 shows the results obtained for all the compared methods, for image sizes 256×256 and SNR = 24 dB. In addition to Normalized error, we also show the PSNR and SSIM of the reconstructed images.

The proposed method obtains better reconstructions in terms of Normalized error, PSNR and SSIM. We also observe that when the number of masks *K* increases, the three methods obtain better reconstructions.

For *K* = 4, the difference in PSNR is approximately 2 dB with the WF, which is the nearest competitor of the GSD-based methods. However, when *K* increases, this difference decreases, and when *K* = 8 the difference with the nearest competitor WF is about 0.8 dB. Regarding running times, the proposed method needs about 1 s when the number of masks is *K* = 8. For the same case, WF and TWF need more than 6 s and TAF and MRTAF more than 3.5 s.

Table 8 and Table 9 show the results obtained for all the compared methods, for image size 512×512 with SNR = 24 dB and SNR = 30 dB, respectively. The proposed method reconstructions have a better Normalized error, PSNR and SSIM with these metrics improving with a higher number of masks.

PhaseSplit and the proposed method result in the same level of reconstruction quality for K=4,8. The proposed method converges 3.5 to 6 times faster than PhaseSplit. For *K* = 4 and SNR = 24 dB there is a 3.5 dB difference with the next best SGD method, TAF. For *K* = 8 there is a 0.85 dB difference with the next best SGD method, WF. In terms of execution time, the proposed method execution time is 2.5 lower than the next best one.

**Figure 6:**
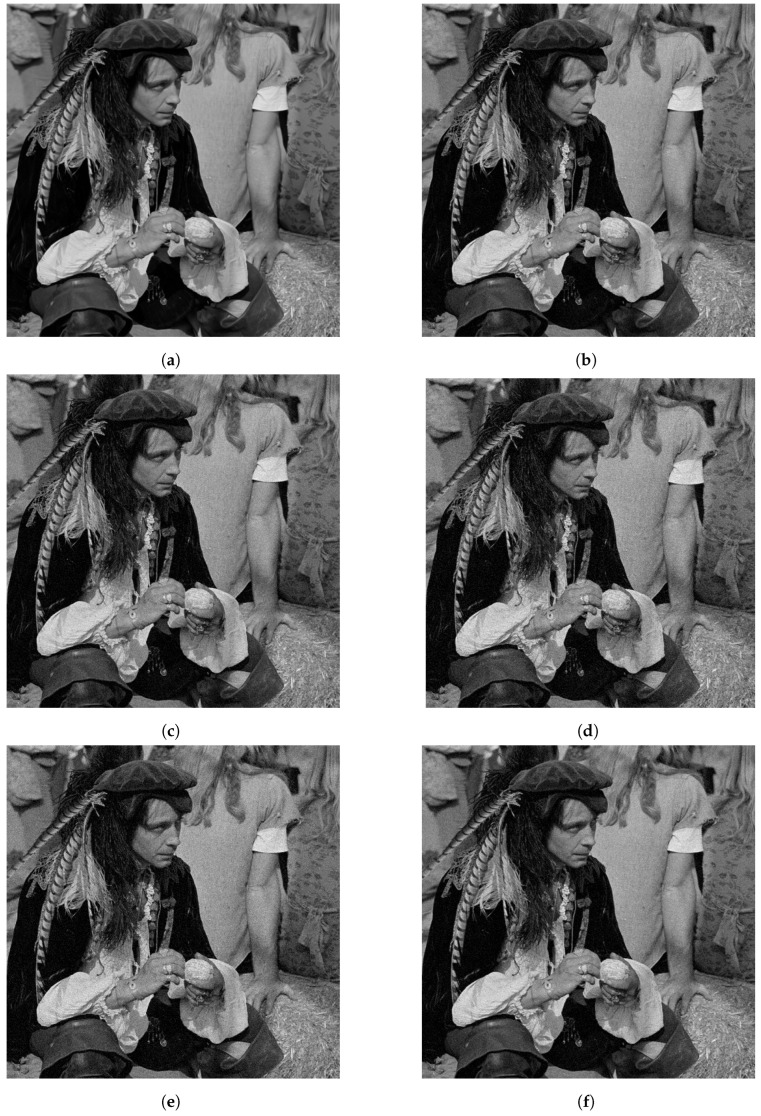
Reconstructions of 512×512 image with SNR = 20 dB for *K* = 4. (**a**) Original Image. (**b**) Reconstruction with Proposed method. (**c**) Reconstruction with WF. (**d**) Reconstruction with TWF. (**e**) Reconstruction with MRTAF. (**f**) Reconstruction with PhaseSplit.

Figure 6 shows an example of the reconstructed images by the compared methods, for image size 512×512, *K* = 4 masks and SNR = 20 dB. Figure 6a shows the ground truth image that we used to generate the observation. Figure 6b shows the reconstruction obtained for the proposed method.

The proposed method recovered most of the details in the image. For instance, see the high-frequencies information on the straw at the bottom-right of the image. See also, the feathers hanging of the man’s hat. Figure 6c,d shows the reconstructions obtained by WF, TWF, MRTAF and PhaseSplit, respectively. These reconstructions are very similar to the ones obtained with the proposed method; however, we can observe that the man’s face in Figure 6c–e, looks noisier than the man’s face in Figure 6b. The reconstruction of PhaseSplit presented in Figure 6f is very similar to the one produced by the proposed method.

### 3.4. Robustness to Number of Masks and Noise Level

**Figure 7:**
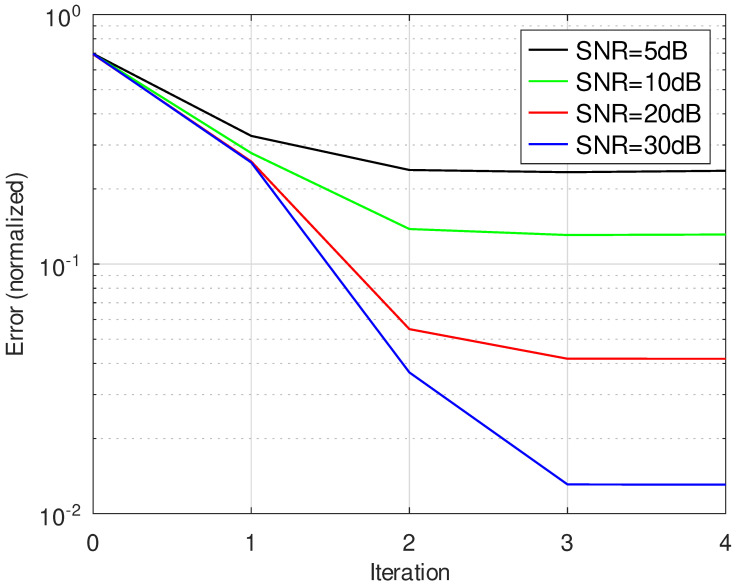
Semilogarithmic plot of normalized error vs. iteration for image size 256×256 and *K* = 8.

The performance of the Proposed method for different levels of noise and number of masks is examined next. Figure 7 shows the effect of noise on the convergence of the proposed method. As expected, we observe that we obtain more error for higher noise levels. However, we observe that for higher noise levels, the proposed method needs less time to converge.

**Figure 8:**
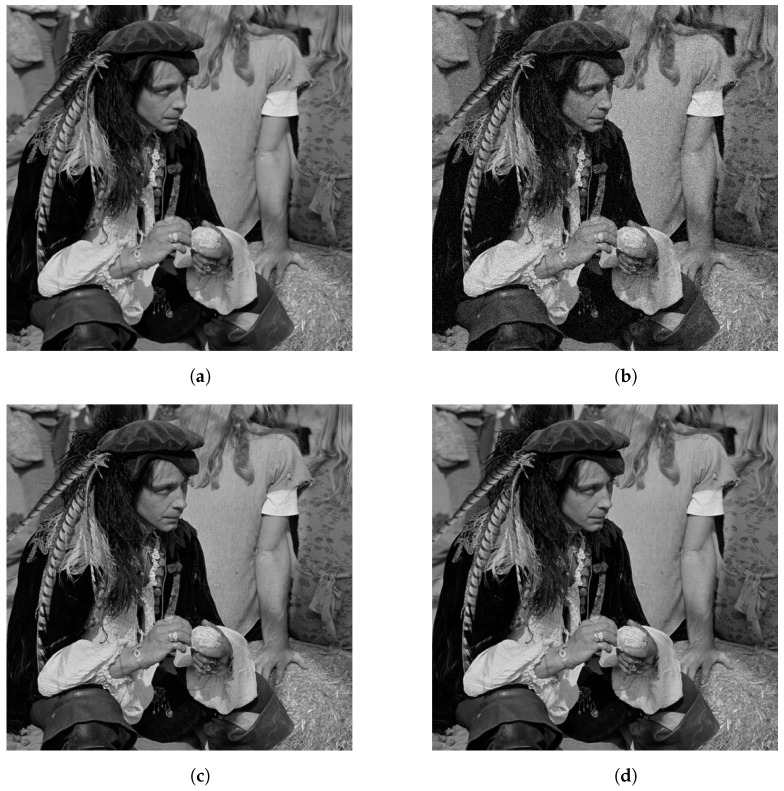
From left to right and top to bottom, original and reconstructions of 512×512 image with *K* = 8 for various noise levels. (**a**) Original Image. (**b**) SNR = 10 dB. (**c**) SNR = 20 dB. (**d**) SNR = 30 dB.

To visualize the effect of various noise levels on the reconstruction, the results for an image, reconstructed with the proposed method, are presented in Figure 8. Figure 8a is the noiseless image. Figure 8b is the reconstructed image with SNR = 10 dB. In this case, there is an obvious effect from the noise in the image quality that can be seen throughout the whole image. Figure 8c,d shows the reconstructed images for SNR = 20 dB and SNR = 30 dB, respectively.

In these cases, the noise effect is less apparent but can be seen as changes in texture, especially on large patches of the same color or texture in the image.

**Figure 9:**
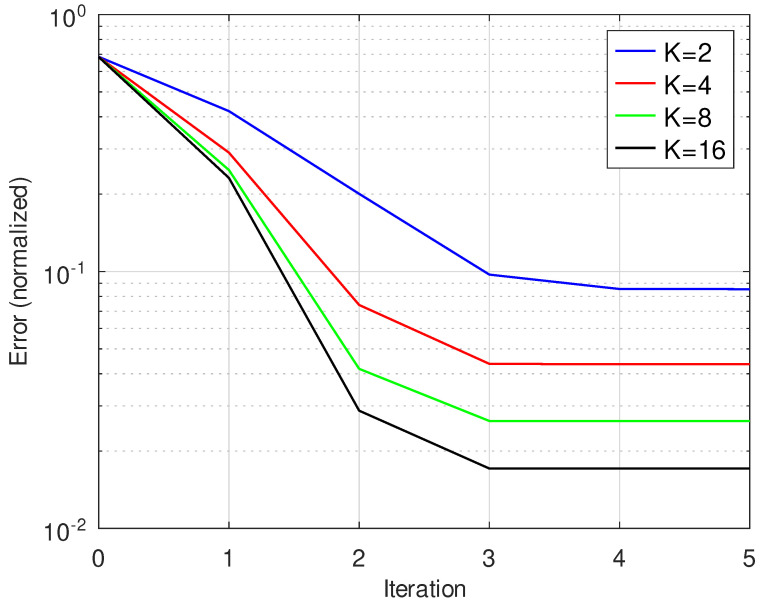
Iteration vs. normalized error for SNR = 24 dB for image size 512×512 and *K* = 2, 4, 8, 16.

Figure 9 shows the performance of the proposed algorithm for different values of *K*. A lower number of masks leads to faster convergence due to lower computational complexity and a higher normalized error.

**Figure 10:**
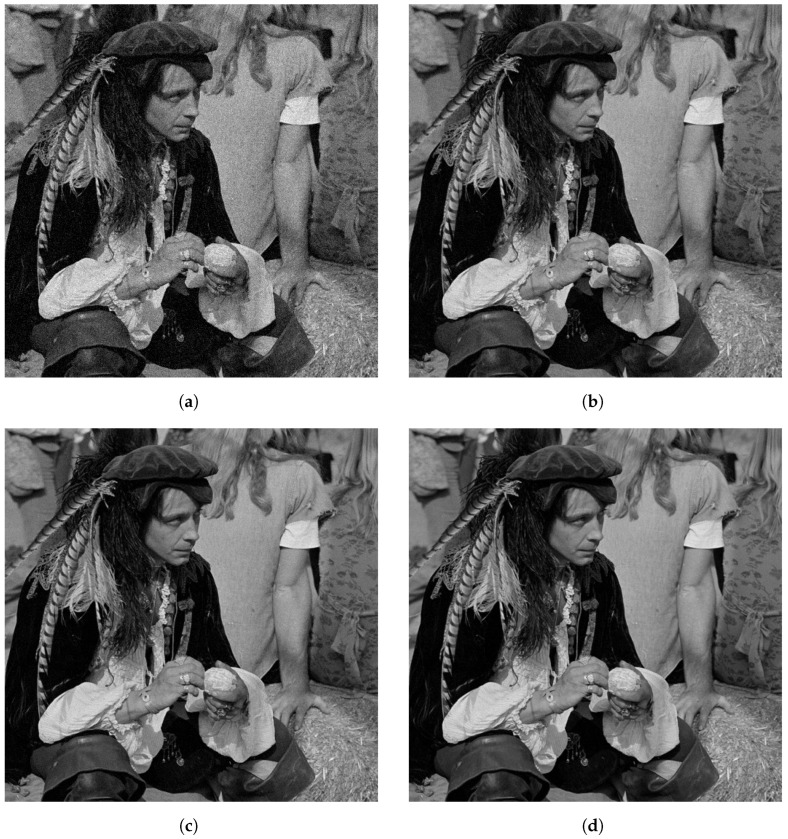
Reconstructions of 512×512 image with SNR = 24 dB for various numbers of masks *K*. (**a**) *K* = 2. (**b**) *K* = 4. (**c**) *K* = 8. (**d**) *K* = 16.

The images in Figure 10 present the corresponding reconstructions for *K* = 2, 4, 8, 16 for a 512×512 image and 20 dB SNR. Figure 10a is the case with *K* = 2 where the effect of noise is most obvious. Figure 10b–d are the cases with *K* = 4, 8 and 16, respectively. In these cases, the reconstruction is better and the effect of noise becomes progressively less apparent with the growing number of masks.

## 4. Discussion

We have presented an analytical method, which outperforms state-of-the-art algorithms for the solution of non-linear quadratic optimization problems associated with Phase Retrieval, when real signals are involved.

Following established results in the literature on the connection between Matrix Completion and Phase Retrieval as well as the uniqueness of the solution under random forward operator conditions [11,22], we have reformulated the original problem into one of alternating optimization with split variables.

While various alternating optimization for Phase Retrieval approaches exist in the literature [30,31] and the formulation considered in this study has also been used in [32], our method differs from other Phase Retrieval solvers, since it introduces an algorithmic step to recombine the split variables and confine the estimated solution to the desired space. This was possible due to a close examination of the relations of the variables involved which allowed us to identify implicit regularizations.

The convergence properties of the algorithm were theoretically examined, in order to establish its applicability for any real signal where it was shown that the algorithm will converge, for some mild initialization conditions.

The presence of noise in the observations is implicitly factored in the statistical uncertainty terms in the theoretical analysis; however, the noise terms were not specifically modeled. An experimental exploration of the effect of AGWN on the method (see Figure 7) showed that the method converges and the output is corrupted according to the noise level. The proposed method performed better than state-of-the-art analytical methods when tested at the same noise level (please see Section 3.3).

Since the algorithm does not use any explicit regularization terms, the only algorithmic parameters that are controllable are the tolerance and maximum iterations of the Conjugate Gradient solver and the number of maximum iterations of Algorithm 1. A higher noise level can allow for the use of higher tolerance or maximum iterations for the GC solver. The proposed algorithm can be implemented on any platform that can support the solution of linear systems via Conjugate Gradient. Since the forward model is based on the Fast Fourier Transform, the only storage requirements are for the masks and split variables, allowing for the handling of large images by standard desktop computers with limited memory.

Our experiments show that given an adequate number of observations, appropriate types of masks and a good initialization, the proposed algorithm can reconstruct any real signal.

However, it must be highlighted that the analysis and implementation of the presented algorithm concerns real signals only. Both the algorithmic implementation and theoretical analysis would be fundamentally different in the case of complex signals. This fact precludes the use of the method in the form presented in this paper by most practical Phase Retrieval applications.

The expansion of this method to its complex signal equivalent and the mapping of the iterations of Algorithm 1 or its complex equivalent onto a Neural Network architecture based on deep unrolling, remain a future research direction.

## 5. Conclusions

In this study, an analytical method to address the problem of Phase Retrieval was developed. The method, which belongs to the family of alternating minimization methods employs an algorithm that estimates two separate variables via convex optimizations iteratively to reach a solution. The discovery of special geometric relations of the split variables brought the introduction of a new algorithmic step, which amounts to the replacement of the variables with their average after the convex estimations at each iteration. A theoretical exploration of the convergence properties of the algorithm in the presence of the recombination step was conducted, showing that the algorithm converges under some mild initialization assumptions. Experiments show that the inclusion of the recombination step leads to significantly faster convergence rates compared to existing analytical methods, for equivalent or improved accuracy, resilience to noise and ability to converge under varying conditions. The presented analysis involves real signals only. The expansion to complex signals and the development of a deep unrolling version of the proposed algorithm are directions of future research.

## Figures and Tables

**Table 1:** URLs of the websites where we downloaded the code for the state-of-the-art methods compared in this work.

Method	URL
WF	https://viterbi-web.usc.edu/ soltanol/PRWF.html (accessed on 20 July 2024)
TAF	https://gangwg.github.io/TAF/codes.html (accessed on 20 July 2024)
TWF	https://yuxinchen2020.github.io/TWF/code.html (accessed on 20 July 2024)

**Table 2:** Comparison of the initialization methods in the noiseless case.

*K*	2			4			8			16		
**Initialization**	**Proposed**	**TWF**	**TAF**	**Proposed**	**TWF**	**TAF**	**Proposed**	**TWF**	**TAF**	**Proposed**	**TWF**	**TAF**
64×64	0.616	1.184	1.172	0.605	1.185	0.673	0.600	0.498	0.426	0.608	0.274	0.292
128×128	0.611	1.217	1.224	0.613	1.219	0.670	0.611	0.501	0.428	0.609	0.350	0.295
256×256	0.631	1.202	1.222	0.630	1.211	0.836	0.632	0.805	0.427	0.631	0.405	0.297
512×512	0.734	1.212	1.221	0.734	1.207	0.915	0.735	0.674	0.427	0.736	0.282	0.296
1024×1024	0.742	1.222	1.220	0.740	1.221	0.644	0.740	0.617	0.428	0.740	0.285	0.298

**Table 3:** Comparison of the initialization methods for SNR = 20 dB.

*K*	2			4			8			16		
**Initialization**	**Proposed**	**TWF**	**TAF**	**Proposed**	**TWF**	**TAF**	**Proposed**	**TWF**	**TAF**	**Proposed**	**TWF**	**TAF**
64×64	0.671	1.221	1.102	0.661	1.056	0.951	0.661	0.774	0.661	0.664	0.956	0.672
128×128	0.673	1.224	1.218	0.675	1.156	1.014	0.671	0.820	0.862	0.685	0.577	0.482
256×256	0.659	1.216	1.204	0.697	1.229	0.974	0.690	1.311	0.761	0.694	0.94	0.624
512×512	0.625	1.222	1.226	0.626	1.198	1.072	0.624	1.372	1.330	0.625	0.87	0.483
1024×1024	0.811	1.223	1.215	0.811	1.198	1.091	0.810	1.38	1.161	0.811	1.24	0.582

**Table 4:** Execution times of the comparison initialization methods in the noiseless case.

*K*	2			4			8			16		
**Initialization**	**WF**	**TWF**	**TAF**	**WF**	**TWF**	**TAF**	**WF**	**TWF**	**TAF**	**WF**	**TWF**	**TAF**
64×64	0.06	0.06	0.09	0.09	0.10	0.12	0.12	0.21	0.20	0.27	0.33	0.35
128×128	0.10	0.19	0.26	0.21	0.28	0.41	0.46	0.61	0.80	0.79	0.94	1.49
256×256	0.51	0.61	0.86	0.87	1.01	1.73	2.09	2.19	2.96	3.88	3.94	7.59
512×512	2.79	3.05	4.81	4.98	4.73	8.87	10.33	11.64	18.65	19.46	24.33	36.62
1024×1024	14.31	15.56	25.31	26.37	30.57	48.99	51.19	59.50	95.22	100.04	117.65	183.56

**Table 5:** Comparison of execution times (in seconds) in the noiseless case. The asterisks (*) denote cases where the reconstruction failed to converge or reach an exact solution.

Initialization Method		Proposed Random				TAF			
***K***	**Size**	**Proposed**	**WF**	**TAF**	**MRTAF**	**Proposed**	**WF**	**TAF**	**MRTAF**
	64×64	0.14	0.72 *	2.67	4.16	0.16	0.71 *	2.59	3.74
	128×128	0.61	3.19 *	13.74	20.17	0.57	3.21 *	13.14	13.34 *
4	256×256	2.04	13.84 *	60.54	80.83	2.41	14.84 *	57.62	84.34
	512×512	15.31	57.54 *	187.34	250.34	14.39	62.12 *	202.24	272.34
	1024×1024	72.41	248.13 *	871.32	1135.12	93.53	239.74 *	876.13	1243.78
	64×64	0.18	1.23 *	1.75	2.49	0.22	1.21	1.76	2.42
	128×128	0.89	5.71 *	9.46	13.09	0.99	5.62	9.45	13.24
8	256×256	5.78	31.09 *	44.54	53.98	4.28	30.79	43.17	52.61
	512×512	26.84	108.34 *	157.72	187.31	28.93	101.13	151.14	191.23
	1024×1024	99.75	464.83 *	627.09	834.12	143.12	611.79	729.32	954.58

**Table 6:** Success rates for the proposed algorithm for real positives and real signals.

Signal Type		$x\in R_{+}^{n}$					$x\in R^{n}$				
**Solver**	**Initialization**	***K* = 2**	***K* = 3**	***K* = 4**	***K* = 5**	***K* = 6**	***K* = 2**	***K *= 3**	***K* = 4**	***K* = 5**	***K* = 6**
Proposed	Random	100%	100%	100%	100%	100%	0%	36%	80%	90%	97%
	TAF	0%	86%	100%	100%	100%	0%	76%	96%	100%	100%
TAF	Random	0%	100%	100%	100%	100%	0%	0%	0%	0%	0%
	TAF	0%	0%	80%	85%	90%	0%	0%	80%	95%	100%
TWF	Random	0%	100%	100%	100%	100%	0%	0%	0%	0%	0%
	TAF	0%	0%	75%	95%	100%	0%	0%	75%	95%	100%
WF	Random	0%	0%	26%	76%	85%	0%	0%	31%	76%	90%
	TAF	0%	0%	24%	75%	85%	0%	0%	30%	75%	90%
MRTAF	Random	0%	100%	100%	100%	100%	0%	0%	0%	0%	0%
	TAF	0%	0%	78%	84%	89%	0%	0%	82%	96%	100%
PhaseSplit	Random	61%	100%	100%	100%	100%	0%	22%	53%	92%	100%
	TAF	68%	100%	100%	100%	100%	0%	31%	94%	98%	100%

**Table 7:** Figures of merit for the three compared methods, for image size 256×256 and SNR = 24 dB.

*K*		2		4		8	
**Initialization**		**Proposed**	**TAF**	**Proposed**	**TAF**	**Proposed**	**TAF**
	Norm. Error	0.086	N/A	0.043	0.043	0.026	0.026
Proposed	PSNR (dB)	25.57	N/A	31.27	31.31	35.19	35.21
	SSIM	0.51	N/A	0.744	0.745	0.874	0.877
	Time (s)	0.45	N/A	0.37	0.82	0.99	1.12
	Norm. Error	N/A	N/A	0.079	0.079	0.041	0.041
WF	PSNR (dB)	N/A	N/A	29.13	29.21	34.41	34.46
	SSIM	N/A	N/A	0.664	0.664	0.853	0.853
	Time (s)	N/A	N/A	6.21	8.01	6.78	6.91
	Norm. Error	0.39	N/A	0.09	0.09	0.054	0.054
TWF	PSNR (dB)	17.49	N/A	27.32	27.38	31.88	31.89
	SSIM	0.172	N/A	0.587	0.587	0.776	0.779
	Time (s)	3.12	N/A	7.72	9.82	6.54	6.67
	Norm. Error	0.36	N/A	0.09	0.09	0.052	0.052
TAF	PSNR (dB)	17.74	N/A	28.61	28.61	32.44	32.46
	SSIM	0.212	N/A	0.620		0.620	0.793
	Time (s)	2.19	N/A	3.57	4.71	3.88	3.79
	Norm. Error	0.38	N/A	0.09	0.09	0.052	0.052
MRTAF	PSNR (dB)	18.00	N/A	28.43	28.46	32.37	37.18
	SSIM	0.181	N/A	0.614	0.614	0.791	0.792
	Time (s)	1.79	N/A	3.87	4.98	3.65	3.71
	Norm. Error	0.28	N/A	0.139	0.139	0.026	0.026
PhaseSplit	PSNR (dB)	19.57	N/A	23.56	22.57	35.16	35.17
	SSIM	0.209	N/A	0.373	0.373	0.874	0.874
	Time (s)	12.31	N/A	4.51	5.55	6.62	6.53

**Table 8:** Comparison of solvers performance, for different *K* and initialization method. The image size is 512×512 and the input SNR is 24 dB.

*K*		2		4		8	
**Initialization**		**Proposed**	**TAF**	**Proposed**	**TAF**	**Proposed**	**TAF**
	Norm. Error	0.085	N/A	0.043	0.043	0.026	0.026
Proposed	PSNR (dB)	27.17	N/A	33.29	33.27	36.92	36.93
	SSIM	0.558	N/A	0.802	0.802	0.913	0.913
	Time (s)	2.53	N/A	3.29	3.42	4.01	4.11
	Norm. Error	N/A	N/A	0.10	0.10	0.041	0.041
WF	PSNR (dB)	N/A	N/A	27.58	27.58	36.06	36.09
	SSIM	N/A	N/A	0.638	0.638	0.897	0.897
	Time (s)	N/A	N/A	18.71	19.12	19.6	20.1
	Norm. Error	0.37	N/A	0.09	0.09	0.055	0.055
TWF	PSNR (dB)	18.171	N/A	29.71	29.73	33.55	33.55
	SSIM	0.14	N/A	0.639	0.639	0.835	0.835
	Time (s)	20.91	N/A	31.51	32.12	19.54	19.61
	Norm. Error	0.35	N/A	0.09	0.09	0.052	0.052
TAF	PSNR (dB)	18.83	N/A	29.81	29.81	34.24	34.24
	SSIM	0.159	N/A	0.671	0.671	0.847	0.847
	Time (s)	8.59	N/A	14.97	15.12	10.46	10.91
	Norm. Error	0.36	N/A	0.09	0.09	0.052	0.052
MRTAF	PSNR (dB)	19.33	N/A	29.78	29.75	34.18	34.24
	SSIM	0.157	N/A	0.671	0.671	0.846	0.846
	Time (s)	8.61	N/A	17.64	16.34	10.94	11.45
	Norm. Error	0.21	N/A	0.043	0.043	0.026	0.026
PhaseSplit	PSNR (dB)	21.76	N/A	33.26	33.24	36.87	36.90
	SSIM	0.244	N/A	0.802	0.802	0.913	0.913
	Time (s)	15.23	N/A	11.77	11.89	24.71	24.34

**Table 9:** Comparison of solvers performance, for different *K* and initialization method. The image size is 512×512 and the input SNR is 30 dB.

*K*		2		4		8	
**Initialization**		**Proposed**	**TAF**	**Proposed**	**TAF**	**Proposed**	**TAF**
	Norm. Error	0.042	N/A	0.021	0.021	0.013	0.013
Proposed	PSNR (dB)	32.67	N/A	38.82	38.84	42.92	42.93
	SSIM	0.834	N/A	0.937	0.937	0.975	0.975
	Time (s)	3.10	N/A	3.98	3.82	6.47	6.78
	Norm. Error	N/A	N/A	0.10	0.10	0.020	0.020
WF	PSNR (dB)	N/A	N/A	26.21	26.18	42.06	42.09
	SSIM	N/A	N/A	0.658	0.659	0.970	0.970
	Time (s)	N/A	N/A	19.71	20.13	34.6	35.1
	Norm. Error	0.35	N/A	0.05	0.05	0.027	0.027
TWF	PSNR (dB)	19.171	N/A	34.41	34.49	39.55	39.55
	SSIM	0.161	N/A	0.854	0.854	0.948	0.948
	Time (s)	21.91	N/A	34.51	36.71	28.54	28.61
	Norm. Error	0.32	N/A	0.044	0.044	0.025	0.025
TAF	PSNR (dB)	19.83	N/A	35.65	35.67	40.12	40.34
	SSIM	0.159	N/A	0.883	0.883	0.954	0.954
	Time (s)	18.59	N/A	10.97	10.64	17.46	17.91
	Norm. Error	0.34	N/A	0.045	0.045	0.026	0.026
MRTAF	PSNR (dB)	19.11	N/A	35.36	35.41	40.01	40.03
	SSIM	0.170	N/A	0.877	0.877	0.953	0.953
	Time (s)	7.98	N/A	19.64	19.45	16.54	16.65
	Norm. Error	0.42	N/A	0.021	0.021	0.014	0.014
PhaseSplit	PSNR (dB)	32.66	N/A	38.79	38.77	41.87	41.90
	SSIM	0.810	N/A	0.937	0.937	0.974	0.974
	Time (s)	12.23	N/A	11.77	11.81	19.71	19.84

## Data Availability

The data and software can be provided by authors upon request.

## Supplementary Materials

The following supporting information can be downloaded at: https://www.mdpi.com/article/10.3390/jimaging10100249/s1. Table S1: Example of evolution of variables after alternatingly estimating a and b. Table S2: Example of evolution of variables at each iteration of the algorithm. The values shown are before averaging the vectors. Figure S1: Values of objective function and normalized norms of vectors d and e after each step of the algorithm. The error norm decreases faster as the algorithm progresses.

## References

[1] Tian L., Waller L. (2015). 3D intensity and phase imaging from light field measurements in an LED array microscope. Optica.

[2] Harrison R.W. (1993). Phase problem in crystallography. J. Opt. Soc. Am. A.

[3] Pfeiffer F., Weitkamp T., Bunk O., David C. (2006). Phase retrieval and differential phase-contrast imaging with low-brilliance X-ray sources. Nat. Phys..

[4] Miao J., Ishikawa T., Johnson B., Anderson E.H., Lai B., Hodgson K.O. (2002). High Resolution 3D X-Ray Diffraction Microscopy. Phys. Rev. Lett..

[5] Miao J., Ishikawa T., Shen Q., Earnest T. (2008). Extending X-ray Crystallography to Allow the Imaging of Noncrystalline Materials, Cells, and Single Protein Complexes. Annu. Rev. Phys. Chem..

[6] Chai A., Moscoso M., Papanicolaou G. (2011). Array imaging using intensity-only measurements. Inverse Probl..

[7] Ahmed A., Recht B., Romberg J.K. (2014). Blind Deconvolution Using Convex Programming. IEEE Trans. Inf. Theory.

[8] Balan R., Casazza P., Edidin D. (2006). On signal reconstruction without phase. Appl. Comput. Harmon. Anal..

[9] Demanet L., Jugnon V. (2017). Convex Recovery From Interferometric Measurements. IEEE Trans. Comput. Imaging.

[10] Fienup C., Dainty J. (1987). Phase retrieval and image reconstruction for astronomy. Image Recover. Theory Appl..

[11] Candès E.J., Strohmer T., Voroninski V. (2011). PhaseLift: Exact and Stable Signal Recovery from Magnitude Measurements via Convex Programming. arXiv.

[12] Candès E.J., Eldar Y.C., Strohmer T., Voroninski V. (2015). Phase Retrieval via Matrix Completion. SIAM Rev..

[13] Gerchberg R., Saxton W.O. (1971). A practical algorithm for the determination of phase from image and diffraction plane pictures. SPIE Milest. Ser. MS.

[14] Maiden A., Rodenburg J.M. (2009). An improved ptychographical phase retrieval algorithm for diffractive imaging. Ultramicroscopy.

[15] Thibault P., Dierolf M., Menzel A., Bunk O., David C., Pfeiffer F. (2008). High-Resolution Scanning X-ray Diffraction Microscopy. Science.

[16] Candes E.J., Wakin M.B. (2008). An Introduction to Compressive Sampling. IEEE Signal Process. Mag..

[17] Candès E.J., Recht B. (2009). Exact Matrix Completion via Convex Optimization. Found. Comput. Math..

[18] Eldar Y.C., Mendelson S. (2014). Phase retrieval: Stability and recovery guarantees. Appl. Comput. Harmon. Anal..

[19] Wang G., Zhang L., Giannakis G.B., Akçakaya M., Chen J. (2018). Sparse Phase Retrieval via Truncated Amplitude Flow. IEEE Trans. Signal Process..

[20] Bandeira A.S., Cahill J., Mixon D.G., Nelson A.A. (2014). Saving phase: Injectivity and stability for phase retrieval. Appl. Comput. Harmon. Anal..

[21] Waldspurger I., d’Aspremont A., Mallat S. (2012). Phase Recovery, MaxCut and Complex Semidefinite Programming. Math. Program..

[22] Candes E.J., Li X., Soltanolkotabi M. (2015). Phase retrieval from coded diffraction patterns. Appl. Comput. Harmon. Anal..

[23] Chen Y., Candés E.J. (2015). Solving Random Quadratic Systems of Equations is Nearly as Easy as Solving Linear Systems. Proceedings of the 28th International Conference on Neural Information Processing Systems—Volume 1.

[24] Bostan E., Soltanolkotabi M., Ren D., Waller L. Accelerated Wirtinger Flow for Multiplexed Fourier Ptychographic Microscopy. Proceedings of the 2018 25th IEEE International Conference on Image Processing (ICIP).

[25] Zhang H., Chi Y., Liang Y. Provable Non-convex Phase Retrieval with Outliers: Median TruncatedWirtinger Flow. Proceedings of the International Conference on Machine Learning.

[26] Cai T., Li X., Ma Z. (2015). Optimal Rates of Convergence for Noisy Sparse Phase Retrieval via Thresholded Wirtinger Flow. Ann. Stat..

[27] Kong L., Yan A. (2023). Robust amplitude method with *L*_1/2_-regularization for compressive phase retrieval. J. Ind. Manag. Optim..

[28] Zhang Q., Liu D., Hu F., Li A., Cheng H. (2021). Median momentum reweighted amplitude flow for phase retrieval with arbitrary corruption. J. Mod. Opt..

[29] Shechtman Y., Beck A., Eldar Y.C. (2014). GESPAR: Efficient phase retrieval of sparse signals. IEEE Trans. Signal Process..

[30] Netrapalli P., Jain P., Sanghavi S. Phase retrieval using alternating minimization. Proceedings of the Advances in Neural Information Processing Systems.

[31] Waldspurger I. (2016). Phase Retrieval With Random Gaussian Sensing Vectors by Alternating Projections. IEEE Trans. Inf. Theory.

[32] Mukherjee S., Shit S., Seelamantula C.S. Phasesplit: A Variable Splitting Framework for Phase Retrieval. Proceedings of the 2018 IEEE International Conference on Acoustics, Speech and Signal Processing (ICASSP).

[33] Bahmani S., Romberg J. (2016). Phase retrieval meets statistical learning theory: A flexible convex relaxation. arXiv.

[34] Goldstein T., Studer C. (2016). PhaseMax: Convex Phase Retrieval via Basis Pursuit. arXiv.

[35] Vaswani N., Nayer S., Eldar Y.C. (2017). Low-Rank Phase Retrieval. IEEE Trans. Signal Process..

[36] Horstmeyer R., Chen R.Y., Ou X., Ames B., Tropp J.A., Yang C. (2015). Solving ptychography with a convex relaxation. New J. Phys..

[37] Wu T.T., Huang C., Gu X., Niu J., Zeng T. (2023). Finding robust minimizer for non-convex phase retrieval. Inverse Probl. Imaging.

[38] Sun J., Qu Q., Wright J. (2016). A geometric analysis of phase retrieval. Proceedings of the 2016 IEEE International Symposium on Information Theory (ISIT).

[39] Schniter P., Rangan S. (2015). Compressive Phase Retrieval via Generalized Approximate Message Passing. IEEE Trans. Signal Process..

[40] Sinha A., Lee J., Li S., Barbastathis G. (2017). Lensless computational imaging through deep learning. Optica.

[41] Deng M., Li S., Zhang Z., Kang I., Fang N.X., Barbastathis G. (2020). On the interplay between physical and content priors in deep learning for computational imaging. Opt. Express.

[42] Metzler C.A., Schniter P., Veeraraghavan A., Baraniuk R.G. (2018). prDeep: Robust Phase Retrieval with Flexible Deep Neural Networks. arXiv.

[43] Jagatap G., Hegde C. (2019). Algorithmic guarantees for inverse imaging with untrained network priors. Proceedings of the 33rd International Conference on Neural Information Processing Systems.

[44] Chen Q., Huang D., Chen R. (2022). Fourier ptychographic microscopy with untrained deep neural network priors. Opt. Express.

[45] Wu X., Wu Z., Shanmugavel S.C., Yu H.Z., Zhu Y. (2022). Physics-informed neural network for phase imaging based on transport of intensity equation. Opt. Express.

[46] Yang Y., Lian Q., Zhang X., Zhang D., Zhang H. (2023). HIONet: Deep priors based deep unfolded network for phase retrieval. Digit. Signal Process..

[47] Zhang J., Xu T., Shen Z., Qiao Y., Zhang Y. (2019). Fourier ptychographic microscopy reconstruction with multiscale deep residual network. Opt. Express.

[48] Rivenson Y., Zhang Y., Günaydın H., Teng D., Ozcan A. (2018). Phase recovery and holographic image reconstruction using deep learning in neural networks. Light. Sci. Appl..

[49] Zhang Y., Noack M.A., Vagovic P., Fezzaa K., Garcia-Moreno F., Ritschel T., Villanueva-Perez P. (2021). PhaseGAN: A deep-learning phase-retrieval approach for unpaired datasets. Opt. Express.

[50] Huang L., Chen H., Liu T., Ozcan A. (2023). Self-supervised learning of hologram reconstruction using physics consistency. Nat. Mach. Intell..

[51] Wang Y., Sun X., Fleischer J.W. When deep denoising meets iterative phase retrieval. Proceedings of the 37th International Conference on Machine Learning.

[52] Hyder R., Cai Z., Asif M.S. (2022). Data-driven illumination patterns for coded diffraction imaging. Sensors.

[53] Wang F., Bian Y., Wang H., Lyu M., Pedrini G., Osten W., Barbastathis G., Situ G. (2020). Phase imaging with an untrained neural network. Light. Sci. Appl..

[54] Song L., Lam E.Y. (2023). Phase retrieval with a dual recursive scheme. Opt. Express.

[55] Wang Z., Zheng S., Ding Z., Guo C. (2024). Dual-constrained physics-enhanced untrained neural network for lensless imaging. J. Opt. Soc. Am. A.

[56] Zhang J., Xu T., Shen Z., Qiao Y., Zhang Y. (2024). ADMM based Fourier phase retrieval with untrained generative prior. J. Comput. Appl. Math..

[57] Wang K., Song L., Wang C., Ren Z., Zhao G., Dou J., Di J., Barbastathis G., Zhou R., Zhao J. (2023). On the use of deep learning for phase recovery. arXiv.

[58] Liu Y., Chen B., Li E., Wang J., Marcelli A., Wilkins S., Ming H., Tian Y., Nugent K., Zhu P. (2008). Phase retrieval in X-ray imaging based on using structured illumination. Phys. Rev. A.

[59] Rodenburg J.M. (2008). Ptychography and related diffractive imaging methods. Adv. Imaging Electron. Phys..

[60] Recht B., Fazel M., Parrilo P.A. (2010). Guaranteed Minimum-Rank Solutions of Linear Matrix Equations via Nuclear Norm Minimization. SIAM Rev..

[61] Barrett R., Berry M., Chan T.F., Demmel J., Donato J., Dongarra J., Eijkhout V., Pozo R., Romine C., Van der Vorst H. (1996). Templates for the solution of linear systems: Building blocks for iterative methods. Math. Comput..

[62] Guerrero A., Pinilla S., Arguello H. (2020). Phase Recovery Guarantees From Designed Coded Diffraction Patterns in Optical Imaging. IEEE Trans. Image Process..

[63] Candès E.J., Li X., Soltanolkotabi M. (2015). Phase Retrieval via Wirtinger Flow: Theory and Algorithms. IEEE Trans. Inf. Theory.

