# Supplementary material for: Variable Splitting and Fusing for Image Phase Retrieval
Source: J Imaging. 2024 Oct 12;10(10):249. doi: 10.3390/jimaging10100249 (PMC11508883; doi:10.3390/jimaging10100249)
Supplement: Supplementary file 1 [file jimaging-10-00249-s001.zip › jimaging-3173117-supplementary.pdf]

Supplementary material for the article Variable Splitting and Fusing for Image Phase Retrieval

S1 Equations at local minima

Consider a linear forward model of the type

$$\mathbf{o} = H\mathbf{x} \tag{S1}$$

with

$$H = \begin{bmatrix} \mathbf{FW}_1 \\ \mathbf{FW}_2 \\ \vdots \\ \mathbf{FW}_L \end{bmatrix}, \tag{S2}$$

where $\mathbf{x} \in \mathcal{R}^N$, $\mathbf{F}$ the $N \times N$ DFT matrix and $\mathbf{W}_l$ the diagonal matrix representing the elementwise product of a vector with N elements randomly picked from the set $[1, -1, i, -i]$.

The observation model for quadratic measurements from this model can be expressed as

$$\mathbf{y} = \overline{H\mathbf{x}} \odot H\mathbf{x}, \tag{S3}$$

where $\overline{H\mathbf{x}}$ is the complex conjugate of $H\mathbf{x}$ and $\odot$ denotes the elementwise product.

Now let

$$\mathbf{y} = \mathbf{H_a b} = Re\{\overline{H\mathbf{a}} \odot H\mathbf{b}\}, \tag{S4}$$

where $\mathbf{H_a}$ is defined in equation (S9) of the main text and $\mathbf{y}$ can be interpreted as the real part of the cross correlation of the modulated $\mathbf{a}$ and $\mathbf{b}$.

The following are true for $\mathbf{H_a b}$, when $\mathbf{a}, \mathbf{b} \in \mathcal{R}^N$:

$$\mathbf{H_a b} = Re\{\overline{H\mathbf{a}} \odot H\mathbf{b}\} = Re\{\overline{\overline{H\mathbf{b}} \odot H\mathbf{a}}\} = \mathbf{H_b a} \tag{S5}$$

$$\mathbf{H_{(a+v)} b} = Re\{(\overline{H\mathbf{a}} + \overline{H\mathbf{v}}) \odot H\mathbf{b}\} = \mathbf{H_a b} + \mathbf{H_v b}. \tag{S6}$$

At each iteration, the vector $\mathbf{a}$ is computed via an Linear Least Squares system solution

$$\mathbf{a} = (\mathbf{H_b^T H_b})^{-1} \mathbf{H_b^T y}. \tag{S7}$$

At this optimal point it holds that

$$\mathbf{H_b^T H_b a} = \mathbf{H_b^T y} = \mathbf{H_b^T H_x x}. \tag{S8}$$

Let $\mathbf{d}$ and $\mathbf{e}$ be the error vectors for $\mathbf{a}$ and $\mathbf{b}$ respectively, such that

$$\mathbf{a} = \mathbf{x} + \mathbf{d}, \tag{S9}$$

$$\mathbf{b} = \mathbf{x} + \mathbf{e}. \tag{S10}$$

From the above

$$\mathbf{H_b^T H_b a} = \mathbf{H_b^T H_x x}, \tag{S11}$$

can be written as

$$\mathbf{H_b^T H_b (x + d)} = \mathbf{H_b^T H_{(b-e)} x} \tag{S12}$$

and after some algebra

$$\mathbf{H_b^T H_b d} = -\mathbf{H_b^T H_x e}. \tag{S13}$$

By the definition of the error vectors, we can write the average of $\mathbf{a}$ and $\mathbf{b}$ as

$$0.5(\mathbf{a} + \mathbf{b}) = \mathbf{x} + 0.5(\mathbf{e} + \mathbf{d}) = \mathbf{x} + 0.5\mathbf{g}, \tag{S14}$$

where $\mathbf{g} = \mathbf{d} + \mathbf{e}$.

Then

$$\mathbf{H}_{\mathbf{b}}^T \mathbf{H}_{\mathbf{b}} \mathbf{d} = -\mathbf{H}_{\mathbf{b}}^T \mathbf{H}_{\mathbf{x}} \mathbf{e} \tag{S15}$$

or

$$\mathbf{H}_{\mathbf{b}}^T \mathbf{H}_{\mathbf{b}} \mathbf{d} = -\mathbf{H}_{\mathbf{b}}^T \mathbf{H}_{(\mathbf{b}-\mathbf{e})} \mathbf{e} \tag{S16}$$

or

$$\mathbf{H}_{\mathbf{b}}^T \mathbf{H}_{\mathbf{b}} \mathbf{d} + \mathbf{H}_{\mathbf{b}}^T \mathbf{H}_{\mathbf{b}} \mathbf{e} = \mathbf{H}_{\mathbf{b}}^T \mathbf{H}_{\mathbf{e}} \mathbf{e} \tag{S17}$$

or

$$\mathbf{H}_{\mathbf{b}}^T \mathbf{H}_{\mathbf{b}} \mathbf{g} = \mathbf{H}_{\mathbf{b}}^T \mathbf{H}_{\mathbf{e}} \mathbf{e}. \tag{S18}$$

Taking the inner product of the vectors of both sides of equation (S18) with $\mathbf{g}$,

$$\mathbf{g}^T \mathbf{H}_{\mathbf{b}}^T \mathbf{H}_{\mathbf{b}} \mathbf{g} = \mathbf{g}^T \mathbf{H}_{\mathbf{b}}^T \mathbf{H}_{\mathbf{e}} \mathbf{e}. \tag{S19}$$

S2 Concentration of mean values

S2.1 Expectation of $\mathbf{g}^T \mathbf{H}_{\mathbf{b}}^T \mathbf{H}_{\mathbf{b}} \mathbf{g}$

From the observation model,

$$\mathbf{H}_{\mathbf{b}} \mathbf{g} = Re\{\overline{H\mathbf{b}} \odot H\mathbf{g}\} = Re\{\overline{H\mathbf{b}} \odot H\}\mathbf{g} \tag{S20}$$

since we assume real input vector signals.

Denoting

$$\mathbf{D}_{\mathbf{b}} = diag(H\mathbf{b}), \tag{S21}$$

we have

$$\mathbf{H}_{\mathbf{b}} = Re\{\overline{\mathbf{D}_{\mathbf{b}}} H\} \tag{S22}$$

or

$$\mathbf{H}_{\mathbf{b}} = \frac{1}{2}(\overline{\mathbf{D}_{\mathbf{b}}} H + \mathbf{D}_{\mathbf{b}} \overline{H}). \tag{S23}$$

Hence

$$\mathbf{H}_{\mathbf{b}}^T \mathbf{H}_{\mathbf{b}} = \frac{1}{4}(H^T \overline{\mathbf{D}_{\mathbf{b}}} + \overline{H}^T \mathbf{D}_{\mathbf{b}})(\overline{\mathbf{D}_{\mathbf{b}}} H + \mathbf{D}_{\mathbf{b}} \overline{H}). \tag{S24}$$

Since the matrix $\mathbf{H}_{\mathbf{b}}$ consists of $1 \times L$ blocks, with each block depending on one of the masks, $\mathbf{W}_l$,

$$\mathbf{H}_{\mathbf{b}}^T \mathbf{H}_{\mathbf{b}} = \frac{1}{4} \sum_{l}^{L} (H^T \overline{\mathbf{D}_{\mathbf{b}}}(l) + \overline{H}^T \mathbf{D}_{\mathbf{b}}(l))(\overline{\mathbf{D}_{\mathbf{b}}} H(l) + \mathbf{D}_{\mathbf{b}} \overline{H}(l)), \tag{S25}$$

where the indices l signify the l-th block of each matrix. We can rewrite the sum as

$$\mathbf{H}_{\mathbf{b}}^T \mathbf{H}_{\mathbf{b}} = \frac{1}{4} \sum_{l}^{L} (S_1(l) + S_2(l) + S_3(l) + S_4(l)), \tag{S26}$$

where we define

$$S_1(l) = \overline{\mathbf{W}_l \mathbf{F}} \mathbf{D}_{\mathbf{b}}(l,l) \overline{\mathbf{D}_{\mathbf{b}}}(l,l) \mathbf{F} \mathbf{W}_l = \overline{\mathbf{W}}_l \sum_{k}^{n} (f_k^T \mathbf{W}_l \mathbf{b}) \overline{(f_k^T \mathbf{W}_l \mathbf{b})} \overline{f}_k f_k^T \mathbf{W}_l, \tag{S27}$$

$$S_2(l) = \overline{\mathbf{W}_l \mathbf{F}} \mathbf{D}_{\mathbf{b}}(l,l) \mathbf{D}_{\mathbf{b}}(l,l) \overline{\mathbf{F} \mathbf{W}_l} = \overline{\mathbf{W}}_l \sum_{k}^{n} (f_k^T \mathbf{W}_l \mathbf{b})(f_k^T \mathbf{W}_l \mathbf{b}) \overline{f}_k \overline{f}_k^T \overline{\mathbf{W}}_l, \tag{S28}$$

$$S_3(l) = \mathbf{W}_l\mathbf{F}\overline{\mathbf{D}_\mathbf{b}}(l,l)\overline{\mathbf{D}_\mathbf{b}}(l,l)\mathbf{F}\mathbf{W}_l = \mathbf{W}_l\sum_k^n \overline{(f_k^T\mathbf{W}_l\mathbf{b})(f_k^T\mathbf{W}_l\mathbf{b})} f_k f_k^T \mathbf{W}_l, \tag{S29}$$

$$S_4(l) = \mathbf{W}_l\mathbf{F}\overline{\mathbf{D}_\mathbf{b}}(l,l)\mathbf{D}_\mathbf{b}(l,l)\overline{\mathbf{F}\mathbf{W}_l} = \mathbf{W}_l\sum_k^n \overline{(f_k^T\mathbf{W}_l\mathbf{b})}(f_k^T\mathbf{W}_l\mathbf{b}) f_k \overline{f}_k^T \overline{\mathbf{W}}_l. \tag{S30}$$

From eq (S26), it follows that

$$\mathbf{g}^T\mathbf{H}_\mathbf{b}^T\mathbf{H}_\mathbf{b}\mathbf{g} = \frac{1}{L}\sum_l^L \begin{bmatrix}\mathbf{g}\\ \mathbf{g}\end{bmatrix}^T \begin{bmatrix} S_1(l) & S_2(l)\\ S_3(l) & S_4(l)\end{bmatrix}\begin{bmatrix}\mathbf{g}\\ \mathbf{g}\end{bmatrix} = \begin{bmatrix}\mathbf{g}\\ \mathbf{g}\end{bmatrix}^T \frac{1}{L}\sum_l^L \begin{bmatrix} S_4(l) & S_3(l)\\ S_2(l) & S_1(l)\end{bmatrix}\begin{bmatrix}\mathbf{g}\\ \mathbf{g}\end{bmatrix} \tag{S31}$$

which is an equation of the form

$$\mathbf{g}^T\mathbf{H}_\mathbf{b}^T\mathbf{H}_\mathbf{b}\mathbf{g} = \begin{bmatrix}\mathbf{g}\\ \mathbf{g}\end{bmatrix}^T \frac{1}{nL}\sum_l^L\sum_k^n \mathbf{M}_{l,k}\begin{bmatrix}\mathbf{g}\\ \mathbf{g}\end{bmatrix} = \tag{S32}$$

$$\begin{bmatrix}\mathbf{g}\\ \mathbf{g}\end{bmatrix}^T \frac{1}{nL}\sum_l^L\sum_k^n \begin{bmatrix}\mathbf{W}_l & \mathbf{0}\\ \mathbf{0} & \overline{\mathbf{W}}_l\end{bmatrix}\begin{bmatrix} \overline{(f_k^T\mathbf{W}_l\mathbf{b})}(f_k^T\mathbf{W}_l\mathbf{b})f_k\overline{f}_k^T & \overline{(f_k^T\mathbf{W}_l\mathbf{b})(f_k^T\mathbf{W}_l\mathbf{b})}f_k f_k^T\\ (f_k^T\mathbf{W}_l\mathbf{b})(f_k^T\mathbf{W}_l\mathbf{b})\overline{f}_k\overline{f}_k^T & (f_k^T\mathbf{W}_l\mathbf{b})\overline{(f_k^T\mathbf{W}_l\mathbf{b})}\overline{f}_k f_k^T\end{bmatrix}\begin{bmatrix}\overline{\mathbf{W}}_l & \mathbf{0}\\ \mathbf{0} & \mathbf{W}_l\end{bmatrix}\begin{bmatrix}\mathbf{g}\\ \mathbf{g}\end{bmatrix} \tag{S33}$$

or

$$\mathbf{g}^T\mathbf{H}_\mathbf{b}^T\mathbf{H}_\mathbf{b}\mathbf{g} = \begin{bmatrix}\mathbf{g}\\ \mathbf{g}\end{bmatrix}^T \frac{1}{nL}\sum_l^L\sum_k^n \begin{bmatrix}\mathbf{W}_l & \mathbf{0}\\ \mathbf{0} & \overline{\mathbf{W}}_l\end{bmatrix}\begin{bmatrix} |f_k^T\mathbf{W}_l\mathbf{b}|^2 f_k\overline{f}_k^T & (\overline{f_k^T\mathbf{W}_l\mathbf{b}})^2 f_k f_k^T\\ (f_k^T\mathbf{W}_l\mathbf{b})^2\overline{f}_k\overline{f}_k^T & |f_k^T\mathbf{W}_l\mathbf{b}|^2\overline{f}_k f_k^T\end{bmatrix}\begin{bmatrix}\overline{\mathbf{W}}_l & \mathbf{0}\\ \mathbf{0} & \mathbf{W}_l\end{bmatrix}\begin{bmatrix}\mathbf{g}\\ \mathbf{g}\end{bmatrix}. \tag{S34}$$

Taking the expectation

$$E\{\mathbf{g}^T\mathbf{H}_\mathbf{b}^T\mathbf{H}_\mathbf{b}\mathbf{g}\} = \begin{bmatrix}\mathbf{g}\\ \mathbf{g}\end{bmatrix}^T E\{\frac{1}{nL}\sum_l^L\sum_k^n \mathbf{M}_{l,k}\}\begin{bmatrix}\mathbf{g}\\ \mathbf{g}\end{bmatrix}. \tag{S35}$$

By definition

$$E\{\frac{1}{n}\sum_k^n \mathbf{M}_{l,k}\} = E\{\begin{bmatrix} S_4(l) & S_3(l)\\ S_2(l) & S_1(l)\end{bmatrix}\} \tag{S36}$$

or

$$E\{\frac{1}{n}\sum_k^n \mathbf{M}_{l,k}\} = E\{\frac{1}{n}\sum_k^n \begin{bmatrix}\mathbf{W}_l & \mathbf{0}\\ \mathbf{0} & \overline{\mathbf{W}}_l\end{bmatrix}\begin{bmatrix} |f_k^T\mathbf{W}_l\mathbf{b}|^2 f_k\overline{f}_k^T & (\overline{f_k^T\mathbf{W}_l\mathbf{b}})^2 f_k f_k^T\\ (f_k^T\mathbf{W}_l\mathbf{b})^2\overline{f}_k\overline{f}_k^T & |f_k^T\mathbf{W}_l\mathbf{b}|^2\overline{f}_k f_k^T\end{bmatrix}\begin{bmatrix}\overline{\mathbf{W}}_l & \mathbf{0}\\ \mathbf{0} & \mathbf{W}_l\end{bmatrix}\}. \tag{S37}$$

From Lemma 3.1 and Lemma 3.2 in [1],

$$E\{\begin{bmatrix} S_4(l) & S_3(l)\\ S_2(l) & S_1(l)\end{bmatrix}\} = \begin{bmatrix}\mathbf{b}\mathbf{b}^T + \|\mathbf{b}\|^2\mathbf{I} & 2\mathbf{b}\mathbf{b}^T\\ 2\mathbf{b}\mathbf{b}^T & \mathbf{b}\mathbf{b}^T + \|\mathbf{b}\|^2\mathbf{I}\end{bmatrix} \tag{S38}$$

and since the expectation is independent of the index l,

$$E\{\frac{1}{nL}\sum_l^L\sum_k^n \mathbf{M}_{l,k}\} = \frac{1}{4}\begin{bmatrix}\mathbf{b}\mathbf{b}^T + \|\mathbf{b}\|^2\mathbf{I} & 2\mathbf{b}\mathbf{b}^T\\ 2\mathbf{b}\mathbf{b}^T & \mathbf{b}\mathbf{b}^T + \|\mathbf{b}\|^2\mathbf{I}\end{bmatrix}. \tag{S39}$$

From eq (S35)

$$E\{\mathbf{g}^T\mathbf{H}_\mathbf{b}^T\mathbf{H}_\mathbf{b}\mathbf{g}\} = \frac{1}{4}\begin{bmatrix}\mathbf{g}\\ \mathbf{g}\end{bmatrix}^T\begin{bmatrix}\mathbf{b}\mathbf{b}^T + \|\mathbf{b}\|^2\mathbf{I} & 2\mathbf{b}\mathbf{b}^T\\ 2\mathbf{b}\mathbf{b}^T & \mathbf{b}\mathbf{b}^T + \|\mathbf{b}\|^2\mathbf{I}\end{bmatrix}\begin{bmatrix}\mathbf{g}\\ \mathbf{g}\end{bmatrix} \tag{S40}$$

and after some algebra

$$E\{\mathbf{g}^T\mathbf{H}_\mathbf{b}^T\mathbf{H}_\mathbf{b}\mathbf{g}\} = 1.5(\mathbf{g}^T\mathbf{b})^2 + 0.5\|\mathbf{g}\|^2\|\mathbf{b}\|^2. \tag{S41}$$

S2.2 Concentration of $\mathbf{g}^T\mathbf{H}_\mathbf{b}^T\mathbf{H}_\mathbf{b}\mathbf{g}$

We seek a bound for the distance of $\mathbf{g}^T\mathbf{H}_\mathbf{b}^T\mathbf{H}_\mathbf{b}\mathbf{g}$ and its expected value.

Lemma 7.4 in [2], asserts that for a sufficiently large number of masks L, that is $L \geq c(\delta)log^3n$, it holds that

$$|\frac{1}{nL}\sum_{l}^{L}\sum_{k}^{n}\mathbf{M}_{l,k} - E\{\frac{1}{nL}\sum_{l}^{L}\sum_{k}^{n}\mathbf{M}_{l,k}\}| \leq \delta, \tag{S42}$$

with probability at least $1-(2L+1)/n^3$, with $\mathbf{M}_{l,k}$ defined in equation (S32).

From corollary 7.5 in [2] and equation (S41) it is straightforward to see that

$$|\mathbf{g}^T\mathbf{H}_\mathbf{b}^T\mathbf{H}_\mathbf{b}\mathbf{g} - 1.5(\mathbf{g}^T\mathbf{b})^2 + 0.5\|\mathbf{g}\|^2\|\mathbf{b}\|^2| \leq \frac{\delta}{2}\|\mathbf{g}\|^2 \tag{S43}$$

with the same probability.

2.3 Expectation of $\mathbf{e}^T\mathbf{H}_\mathbf{e}^T\mathbf{H}_\mathbf{b}\mathbf{g}$

By definition

$$\mathbf{H}_\mathbf{b}\mathbf{g} = Re\{\overline{H\mathbf{b}} \odot H\mathbf{g}\} = Re\{\overline{H\mathbf{b}} \odot H\}\mathbf{g}, \tag{S44}$$

$$\mathbf{D}_\mathbf{b} = diag(H\mathbf{b}), \tag{S45}$$

$$\mathbf{H}_\mathbf{b} = Re\{\overline{\mathbf{D}_\mathbf{b}}H\}, \tag{S46}$$

or

$$\mathbf{H}_\mathbf{b} = \frac{1}{2}(\overline{\mathbf{D}_\mathbf{b}}H + \mathbf{D}_\mathbf{b}\overline{H}). \tag{S47}$$

Hence

$$\mathbf{H}_\mathbf{e}^T\mathbf{H}_\mathbf{b} = \frac{1}{4}(H^T\overline{\mathbf{D}_\mathbf{e}} + \overline{H}^T\mathbf{D}_\mathbf{e})(\overline{\mathbf{D}_\mathbf{b}}H + \mathbf{D}_\mathbf{b}\overline{H}). \tag{S48}$$

Since the matrices $\mathbf{H}_\mathbf{e}, \mathbf{H}_\mathbf{b}$ consist of $1 \times L$ blocks, with each block depending on one of the masks, $\mathbf{W}_l$,

$$\mathbf{H}_\mathbf{e}^T\mathbf{H}_\mathbf{b} = \frac{1}{4}\sum_{l}^{L}(H^T\overline{\mathbf{D}_\mathbf{e}}(l) + \overline{H}^T\mathbf{D}_\mathbf{e}(l))(\overline{\mathbf{D}_\mathbf{b}}H(l) + \mathbf{D}_\mathbf{b}\overline{H}(l)), \tag{S49}$$

where the indices l signify the l-th block of each matrix. We can rewrite the sum as

$$\mathbf{H}_\mathbf{e}^T\mathbf{H}_\mathbf{b} = \frac{1}{4}\sum_{l}^{L}(T_1(l) + T_2(l) + T_3(l) + T_4(l)) \tag{S50}$$

where we define

$$T_1(l) = \overline{\mathbf{W}_l\mathbf{F}}\mathbf{D}_\mathbf{e}(l,l)\overline{\mathbf{D}_\mathbf{b}}(l,l)\mathbf{F}\mathbf{W}_l = \overline{\mathbf{W}}_l\sum_{k}^{n}(f_k^T\mathbf{W}_l\mathbf{e})\overline{(f_k^T\mathbf{W}_l\mathbf{b})}\overline{f}_kf_k^T\mathbf{W}_l, \tag{S51}$$

$$T_2(l) = \overline{\mathbf{W}_l\mathbf{F}}\mathbf{D}_\mathbf{e}(l,l)\mathbf{D}_\mathbf{b}(l,l)\overline{\mathbf{F}\mathbf{W}_l} = \overline{\mathbf{W}}_l\sum_{k}^{n}(f_k^T\mathbf{W}_l\mathbf{e})(f_k^T\mathbf{W}_l\mathbf{b})\overline{f}_k\overline{f}_k^T\overline{\mathbf{W}}_l, \tag{S52}$$

$$T_3(l) = \mathbf{W}_l\mathbf{F}\overline{\mathbf{D}_\mathbf{e}}(l,l)\overline{\mathbf{D}_\mathbf{b}}(l,l)\mathbf{F}\mathbf{W}_l = \mathbf{W}_l\sum_{k}^{n}\overline{(f_k^T\mathbf{W}_l\mathbf{e})(f_k^T\mathbf{W}_l\mathbf{b})}f_kf_k^T\mathbf{W}_l, \tag{S53}$$

$$T_4(l) = \mathbf{W}_l\mathbf{F}\overline{\mathbf{D}_\mathbf{e}}(l,l)\mathbf{D}_\mathbf{b}(l,l)\overline{\mathbf{F}\mathbf{W}_l} = \mathbf{W}_l\sum_{k}^{n}\overline{(f_k^T\mathbf{W}_l\mathbf{e})}(f_k^T\mathbf{W}_l\mathbf{b})f_k\overline{f}_k^T\overline{\mathbf{W}}_l. \tag{S54}$$

From eq ($s26$), it follows that

$$\mathbf{e}^T\mathbf{H_e}^T\mathbf{H_b}\mathbf{g} = \frac{1}{4L}\sum_{l}^{L}\begin{bmatrix}\mathbf{e}\\ \mathbf{e}\end{bmatrix}^T\begin{bmatrix}T_1(l) & T_2(l)\\ T_3(l) & T_4(l)\end{bmatrix}\begin{bmatrix}\mathbf{g}\\ \mathbf{g}\end{bmatrix} = \begin{bmatrix}\mathbf{e}\\ \mathbf{e}\end{bmatrix}^T\frac{1}{4L}\sum_{l}^{L}\begin{bmatrix}T_4(l) & T_3(l)\\ T_2(l) & T_1(l)\end{bmatrix}\begin{bmatrix}\mathbf{g}\\ \mathbf{g}\end{bmatrix} \tag{S55}$$

which is an equation of the form

$$\mathbf{e}^T\mathbf{H_e}^T\mathbf{H_b}\mathbf{g} = \begin{bmatrix}\mathbf{e}\\ \mathbf{e}\end{bmatrix}^T\frac{1}{4nL}\sum_{l}^{L}\sum_{k}^{n}\mathbf{K}_{l,k}\begin{bmatrix}\mathbf{g}\\ \mathbf{g}\end{bmatrix}, \tag{S56}$$

expanding to

$$\mathbf{e}^T\mathbf{H_e}^T\mathbf{H_b}\mathbf{g} = \begin{bmatrix}\mathbf{e}\\ \mathbf{e}\end{bmatrix}^T\frac{1}{4nL}\sum_{l}^{L}\sum_{k}^{n}\begin{bmatrix}\mathbf{W}_l & \mathbf{0}\\ \mathbf{0} & \overline{\mathbf{W}_l}\end{bmatrix}\begin{bmatrix}\overline{(f_k^T\mathbf{W}_l\mathbf{e})}(f_k^T\mathbf{W}_l\mathbf{b})f_k\overline{f}_k^T & \overline{(f_k^T\mathbf{W}_l\mathbf{e})(f_k^T\mathbf{W}_l\mathbf{b})}f_kf_k^T\\ (f_k^T\mathbf{W}_l\mathbf{e})(f_k^T\mathbf{W}_l\mathbf{b})\overline{f}_k\overline{f}_k^T & (f_k^T\mathbf{W}_l\mathbf{e})\overline{(f_k^T\mathbf{W}_l\mathbf{b})}\overline{f}_kf_k^T\end{bmatrix}\begin{bmatrix}\overline{\mathbf{W}_l} & \mathbf{0}\\ \mathbf{0} & \mathbf{W}_l\end{bmatrix}\begin{bmatrix}\mathbf{g}\\ \mathbf{g}\end{bmatrix}. \tag{S57}$$

Taking the expectation

$$E\{\mathbf{e}^T\mathbf{H_e}^T\mathbf{H_b}\mathbf{g}\} = \begin{bmatrix}\mathbf{e}\\ \mathbf{e}\end{bmatrix}^T E\{\frac{1}{4nL}\sum_{l}^{L}\sum_{k}^{n}\mathbf{K}_{l,k}\}\begin{bmatrix}\mathbf{g}\\ \mathbf{g}\end{bmatrix}. \tag{S58}$$

By definition

$$E\{\frac{1}{n}\sum_{k}^{n}\mathbf{K}_{l,k}\} = E\{\begin{bmatrix}T_4(l) & T_3(l)\\ T_2(l) & T_1(l)\end{bmatrix}\} \tag{S59}$$

or

$$E\{\frac{1}{n}\sum_{k}^{n}\mathbf{K}_{l,k}\} = E\{\frac{1}{n}\sum_{k}^{n}\begin{bmatrix}\mathbf{W}_l & \mathbf{0}\\ \mathbf{0} & \overline{\mathbf{W}_l}\end{bmatrix}\begin{bmatrix}\overline{(f_k^T\mathbf{W}_l\mathbf{e})}(f_k^T\mathbf{W}_l\mathbf{b})f_k\overline{f}_k^T & \overline{(f_k^T\mathbf{W}_l\mathbf{e})(f_k^T\mathbf{W}_l\mathbf{b})}f_kf_k^T\\ (f_k^T\mathbf{W}_l\mathbf{e})(f_k^T\mathbf{W}_l\mathbf{b})\overline{f}_k\overline{f}_k^T & (f_k^T\mathbf{W}_l\mathbf{e})\overline{(f_k^T\mathbf{W}_l\mathbf{b})}\overline{f}_kf_k^T\end{bmatrix}\begin{bmatrix}\overline{\mathbf{W}_l} & \mathbf{0}\\ \mathbf{0} & \mathbf{W}_l\end{bmatrix}.\} \tag{S60}$$

We now seek closed form solutions for the expectation of the terms $T_1(l), T_2(l), T_3(l), T_4(l)$ (in the following we omit the index l for brevity, nonetheless the result should be the same for each l, since the statistics of the masks $\mathbf{W}_l$ are the same).

$$T_1 = \overline{\mathbf{W}}\overline{\mathbf{F}}\mathbf{D_e}\overline{\mathbf{D_b}}\mathbf{F}\mathbf{W}, \tag{S61}$$

$$T_2 = \overline{\mathbf{W}}\overline{\mathbf{F}}\mathbf{D_e}\mathbf{D_b}\overline{\mathbf{F}}\overline{\mathbf{W}}, \tag{S62}$$

$$T_3 = \mathbf{W}\mathbf{F}\overline{\mathbf{D_e}}\overline{\mathbf{D_b}}\mathbf{F}\mathbf{W}, \tag{S63}$$

$$T_4 = \mathbf{W}\mathbf{F}\overline{\mathbf{D_e}}\mathbf{D_b}\overline{\mathbf{F}}\overline{\mathbf{W}}. \tag{S64}$$

Following a similar methodology as in Lemma 3.1 and Lemma 3.2 from [1], we have for T1

$$T_1 = \overline{\mathbf{W}}\{\sum_{k=1}^{N}\mathbf{D_e}(k,k)\overline{\mathbf{D_b}}(k,k)\overline{f}_kf_k^T\}\mathbf{W} \tag{S65}$$

or for each element

$$T_1(p,q) = \frac{1}{n}\sum_{k=1}^{N}(\sum_{t=1}^{N}\omega^{-(k-1)(t-1)}w_te_t)(\sum_{s=1}^{N}\omega^{(k-1)(s-1)}\overline{w_s}b_s)\omega^{(k-1)(p-1)}\overline{w_p}\omega^{-(k-1)(q-1)}w_q \tag{S66}$$

or

$$T_1(p,q) = \frac{1}{n}\sum_{k=1}^{N}\sum_{s=1}^{N}\sum_{t=1}^{N}\omega^{(k-1)(p-q+s-t)}\overline{w_p}w_q\overline{w_s}w_te_tb_s, \tag{S67}$$

$$T_1(p,q) = \sum_{s=1}^{N}\sum_{t=1}^{N} \overline{w_p} w_q \overline{w_s} w_t e_t b_s \frac{1}{n} \sum_{k=1}^{N} \omega^{(k-1)(p-q+s-t)}. \tag{S68}$$

The terms of the rightmost sum contribute to a nonzero value when $p+s-(q+t)$ is a multiple of n. Denoting congruence as $p+s \stackrel{N}{=} q+t$, the expected value of T_1, is

$$E\{T_1(p,q)\} = \sum_{s=1}^{N}\sum_{t=1}^{N} E\{\overline{w_p} w_q \overline{w_s} w_t\} e_t b_s (1|\{p+s \stackrel{N}{=} q+t\}). \tag{S69}$$

By definition, the coded diffraction patterns [1] statistics must obey the constraints

$$E\{w\} = 0, \tag{S70}$$

$$E\{w^2\} = 0, \tag{S71}$$

$$E\{|w|^4\} = 2E\{|w|^2\}, \tag{S72}$$

and by definition

$$E\{|w|^2\} = 1. \tag{S73}$$

From the above, for the diagonal terms of T_1 $(p=q)$, $E\{\overline{w_p} w_p \overline{w_s} w_t\} = 0$, unless $s=t$ and thus

$$E\{T_1(p,p)\} = \sum_{s=1}^{N}\sum_{t=1}^{N} E\{\overline{w_p} w_p \overline{w_s} w_t\} e_t b_s (1|\{p+s \stackrel{N}{=} q+t\}) \tag{S74}$$

becomes

$$E\{T_1(p,p)\} = \sum_{s=1}^{N} E\{|w_p|^2 |w_s|^2\} e_s b_s (1|\{p+s \stackrel{N}{=} q+t\}). \tag{S75}$$

This sum can be split in two parts, one for $p=s$ and the remaining terms, thus

$$E\{T_1(p,p)\} = E\{|w_p|^4\} e_p b_p + \sum_{s\neq p}^{N} E\{|w_p|^2 |w_s|^2\} e_s b_s = 2 e_p b_p + \sum_{s\neq p}^{N} e_s b_s. \tag{S76}$$

Finally

$$E\{T_1(p,p)\} = e_p b_p + \mathbf{e}^{\mathbf{T}}\mathbf{b}. \tag{S77}$$

For the off diagonal terms $E\{\overline{w_p} w_q \overline{w_s} w_t\} = 0$, unless $s=q,\ t=p$ from which we have

$$E\{T_1(p,q)\} = \sum_{s=1}^{N}\sum_{t=1}^{N} E\{\overline{w_p} w_q \overline{w_s} w_t\} e_t b_s (1|\{p+s \stackrel{N}{=} q+t\}) = e_p b_q. \tag{S78}$$

Combining the above,

$$E\{T_1\} = \mathbf{e}\mathbf{b}^{\mathbf{T}} + (\mathbf{e}^{\mathbf{T}}\mathbf{b})\mathbf{I}. \tag{S79}$$

Following a similar method, we have for T_2

$$E\{T_2(p,q)\} = \sum_{s=1}^{N}\sum_{t=1}^{N} E\{\overline{w_p}\,\overline{w_q} w_s w_t\} e_t b_s (1|\{p+q \stackrel{N}{=} s+t\}). \tag{S80}$$

For the diagonal terms $(p=q)$, $E\{\overline{w_p}\,\overline{w_p} w_s w_t\} = 0$, unless $p=s=t$, from which we get

$$E\{T_2(p,p)\} = 2 e_q b_q. \tag{S81}$$

For the off-diagonal terms, $E\{\overline{w_p w_q} w_s w_t\} = 0$, unless $p = s$ and $q = t$ or $p = t$ and $q = s$, thus

$$E\{T_2(p,q)\} = e_p b_q + b_p e_q \tag{S82}$$

and overall

$$E\{T_2\} = \mathbf{e}\mathbf{b}^{\mathbf{T}} + \mathbf{b}\mathbf{e}^{\mathbf{T}}. \tag{S83}$$

For T_3,

$$E\{T_3(p,q)\} = \sum_{s=1}^{N}\sum_{t=1}^{N} E\{\overline{w_s w_t} w_p w_q\} e_t b_s (1|\{p + q \overset{N}{=} s + t\}) \tag{S84}$$

and similar to T_2,

$$E\{T_3\} = \mathbf{b}\mathbf{e}^{\mathbf{T}} + \mathbf{e}\mathbf{b}^{\mathbf{T}}. \tag{S85}$$

Finally, for T_4

$$E\{T_4(p,q)\} = \sum_{s=1}^{N}\sum_{t=1}^{N} E\{w_p \overline{w_q} w_s \overline{w_t}\} e_t b_s (1|\{s + p \overset{N}{=} t + q\}). \tag{S86}$$

Similar to T_1 for diagonal terms the non zero contributing factors are for $s = t$,

$$E\{T_4(p,p)\} = \sum_{s=1}^{N} E\{|w_p|^2 |w_s|^2\} e_s b_s (1|\{p + s \overset{N}{=} q + t\}) \tag{S87}$$

or

$$E\{T_4(p,p)\} = E\{|w_p|^4\} e_p b_p + \sum_{s \neq p}^{N} E\{|w_p|^2 |w_s|^2\} e_s b_s = 2 e_p b_p + \sum_{s \neq p}^{N} e_s b_s \tag{S88}$$

and for the off diagonal the non zero contributing factors are for $p = t, \ s = q$,

$$E\{T_4(p,q)\} = \sum_{s=1}^{N}\sum_{t=1}^{N} E\{\overline{w_p} w_q \overline{w_s} w_t\} e_t b_s (1|\{p + s \overset{N}{=} q + t\}) = e_p b_q \tag{S89}$$

,

thus

$$E\{T_4\} = \mathbf{e}\mathbf{b}^{\mathbf{T}} + (\mathbf{e}^{\mathbf{T}}\mathbf{b})\mathbf{I}. \tag{S90}$$

Combining the above, we have from equations, (S58) and (S59),

$$E\{\mathbf{e}^T \mathbf{H}_{\mathbf{e}}^T \mathbf{H}_{\mathbf{b}} \mathbf{g}\} = \begin{bmatrix} \mathbf{e} \\ \mathbf{e} \end{bmatrix}^T \frac{1}{4L} \sum_{l}^{L} E\{\begin{bmatrix} T_4(l) & T_3(l) \\ T_2(l) & T_1(l) \end{bmatrix}\} \begin{bmatrix} \mathbf{g} \\ \mathbf{g} \end{bmatrix} \tag{S91}$$

or

$$E\{\mathbf{e}^T \mathbf{H}_{\mathbf{e}}^T \mathbf{H}_{\mathbf{b}} \mathbf{g}\} = \begin{bmatrix} \mathbf{e} \\ \mathbf{e} \end{bmatrix}^T \frac{1}{4} \begin{bmatrix} \mathbf{e}\mathbf{b}^{\mathbf{T}} + (\mathbf{e}^{\mathbf{T}}\mathbf{b})\mathbf{I} & \mathbf{b}\mathbf{e}^{\mathbf{T}} + \mathbf{e}\mathbf{b}^{\mathbf{T}} \\ \mathbf{b}\mathbf{e}^{\mathbf{T}} + \mathbf{e}\mathbf{b}^{\mathbf{T}} & \mathbf{e}\mathbf{b}^{\mathbf{T}} + (\mathbf{e}^{\mathbf{T}}\mathbf{b})\mathbf{I} \end{bmatrix} \begin{bmatrix} \mathbf{g} \\ \mathbf{g} \end{bmatrix}. \tag{S92}$$

Finally,

$$E\{\mathbf{e}^T \mathbf{H}_{\mathbf{e}}^T \mathbf{H}_{\mathbf{b}} \mathbf{g}\} = \|\mathbf{e}\|^2 (\mathbf{b}^T \mathbf{g}) + (\mathbf{e}^T \mathbf{g})(\mathbf{b}^T \mathbf{e}). \tag{S93}$$

2.4 Concentration of $\mathbf{e}^T\mathbf{H}_\mathbf{e}^T\mathbf{H}_\mathbf{b}\mathbf{g}$

For a sufficiently large number of masks L, that is $L \geq c(\delta)log^3 n$, it holds that

$$|\frac{1}{nL}\sum_l^L\sum_k^n \mathbf{K}_{l,k} - E\{\frac{1}{nL}\sum_l^L\sum_k^n \mathbf{K}_{l,k}\}| \leq \delta \tag{S94}$$

with probability at least $1-(2L+1)/n^3$.

This result can be obtained with a small modification of the proof of Lemma 7.4 in [2].

Specifically, define,

$$\mathbf{Q} = \frac{1}{nL}\sum_l^L\sum_k^n \mathbf{K}_{l,k} \tag{S95}$$

or

$$\mathbf{Q} = \frac{1}{nL}\sum_l^L\sum_k^N \begin{bmatrix} \mathbf{W}_l & \mathbf{0} \\ \mathbf{0} & \overline{\mathbf{W}_l} \end{bmatrix} \begin{bmatrix} \overline{(f_k^T\mathbf{W}_l\mathbf{c})}(f_k^T\mathbf{W}_l\mathbf{a})f_k\overline{f}_k^T & \overline{(f_k^T\mathbf{W}_l\mathbf{c})(f_k^T\mathbf{W}_l\mathbf{a})}f_kf_k^T \\ (f_k^T\mathbf{W}_l\mathbf{c})(f_k^T\mathbf{W}_l\mathbf{a})\overline{f}_k\overline{f}_k^T & (f_k^T\mathbf{W}_l\mathbf{c})\overline{(f_k^T\mathbf{W}_l\mathbf{a})}\overline{f}_kf_k^T \end{bmatrix} \begin{bmatrix} \overline{\mathbf{W}_l} & \mathbf{0} \\ \mathbf{0} & \mathbf{W}_l \end{bmatrix} \tag{S96}$$

and

$$\tilde{\mathbf{Q}} = \frac{1}{nL}\sum_l^L\sum_k^n \mathbf{K}_{l,k}\mathbf{1}_{\{|\overline{f}_k^T\mathbf{W}_l\mathbf{a}|\leq\sqrt{2R\log n},\ |\overline{f}_k^T\mathbf{W}_l\mathbf{c}|\leq\sqrt{2R\log n}\}}, \tag{S97}$$

for a positive scalar R.

Setting the events

$$E_1(R) = \{|\tilde{\mathbf{Q}} - E\{\mathbf{Q}\}| \leq \epsilon\}, \tag{S98}$$

$$E_2(R) = \{\tilde{\mathbf{Q}} = \mathbf{Q}\}, \tag{S99}$$

$$E_3(R) = \cap_{k,l}\{|\overline{f}_k^T\mathbf{W}_l\mathbf{a}| \leq \sqrt{2R\log n},\ |\overline{f}_k^T\mathbf{W}_l\mathbf{c}| \leq \sqrt{2R\log n}\}, \tag{S100}$$

$$E = \{|\mathbf{Q} - E\{\mathbf{Q}\}| \leq \epsilon\}. \tag{S101}$$

If $|\overline{f}_k^T\mathbf{W}_l\mathbf{a}| \leq \sqrt{2R\log n}$ and $|\overline{f}_k^T\mathbf{W}_l\mathbf{c}| \leq \sqrt{2R\log n},\ \forall\ k,l$, then $E_3 \subset E_2$. Denoting $P(E^c)$ the complementary event of E,

$$P(E^c) \leq P(E_1^c \cup E_2^c) \leq P(E_1^c) + P(E_2^c) \leq P(E_1^c) + P(E_3^c) \tag{S102}$$

or

$$P(E^c) \leq P(E_1^c) + \sum_k\sum_l P(|\overline{f}_k^T\mathbf{W}_l\mathbf{a}| > \sqrt{2R\log n} \cap |\overline{f}_k^T\mathbf{W}_l\mathbf{c}| > \sqrt{2R\log n}). \tag{S103}$$

Statistical independence is a valid assumption, since the vectors $\mathbf{e}$ and $\mathbf{b}$, do not converge as the algorithm progresses, thus

$$P(E^c) \leq P(E_1^c) + \sum_k\sum_l P(|\overline{f}_k^T\mathbf{W}_l\mathbf{a}| > \sqrt{2R\log n})P(|\overline{f}_k^T\mathbf{W}_l\mathbf{c}| > \sqrt{2R\log n}) \tag{S104}$$

from which

$$P(E^c) \leq P(E_1^c) + \sum_k\sum_l P(|\overline{f}_k^T\mathbf{W}_l\mathbf{a}| > \sqrt{2R\log n}) \tag{S105}$$

and combining with the Hoeffding inequality

$$P(E^c) \leq P(E_1^c) + 2Ln^{1-R}. \tag{S106}$$

With a modification of Lemma 3.9 in [1], we have $P(E_1^c) \leq 1/n^3$, when $L \geq c(R)\log^3 n$.

From the above,

$$P(E^c) \leq 1 - (2L+1)/n^3. \tag{S107}$$

From equation (S93) and inequality (S94) we have that

$$|\mathbf{e}^T \mathbf{H}_{\mathbf{e}}^T \mathbf{H}_{\mathbf{b}} \mathbf{g} - \|\mathbf{e}\|^2 (\mathbf{b}^T \mathbf{g}) + (\mathbf{e}^T \mathbf{g})(\mathbf{b}^T \mathbf{e})| \leq \delta \|\mathbf{g}\| \|\mathbf{e}\|, \tag{S108}$$

with probability at least $1 - (2L+1)/n^3$.

S3 Computational complexity of proposed method

Algorithm 1 involves the solution of a linear system of equations of the form

$$(\mathbf{H}_{\mathbf{a}^{(n-1)}})^T \mathbf{H}_{\mathbf{a}^{(n-1)}} \mathbf{b} = (\mathbf{H}_{\mathbf{a}^{(n-1)}})^T \mathbf{y}. \tag{S109}$$

Since the left side linear operator of equation (S109) consists of the product of a matrix with its transpose, it is symmetric and positive definite. The Conjugate Gradient method [3] can thus be used to calculate the solution of the system.

The most computationally expensive step of the Conjugate Gradient method is the application of the linear operator to an estimate variable $\mathbf{z}$. In the case of Algorithm 1, the estimate variable are of size N. The matrix $(\mathbf{H}_{\mathbf{a}^{(n-1)}})^T \mathbf{H}_{\mathbf{a}^{(n-1)}}$ has a special structure, since it can be factorized with factors that contain the Discrete and inverse Discrete Fourier Transform Matrix.

The calculation of $\mathbf{H}_{\mathbf{a}^{(n-1)}} \mathbf{z}$ amounts to the elementwise product of the vectors $\mathbf{a}^{(n-1)}$ and $\mathbf{z}$ with the K different masks and tranforming them with the inverse Discrete Fourier transform and the Discrete Fourier transform respectively, before outputing the real part of their collated elementwise product. Since the computational complexity of the Fourier transform is $\mathcal{O}(NlogN)$, the overall complexity of this operation is $\mathcal{O}(2KNlogN + 3KN)$.

To apply the $(\mathbf{H}_{\mathbf{a}^{(n-1)}})^T$ operator, first the elementwise product of the modulated and transformed $\mathbf{a}^{(n-1)}$ for all K masks with the output of $\mathbf{H}_{\mathbf{a}^{(n-1)}} \mathbf{z}$. Then, the sum of the outcome of this operation after a Discrete Fourier Transformation and elementwise multiplication with the mask elements is computed. Overall, this operation also has a complexity of $\mathcal{O}(2KNlogN + 3KN)$.

Since the modulated and transformed vector $\mathbf{H}_{\mathbf{a}^{(n-1)}}$ is involved in both operators, it has to be calculated only once. Thus the calculation $(\mathbf{H}_{\mathbf{a}^{(n-1)}})^T \mathbf{H}_{\mathbf{a}^{(n-1)}} \mathbf{z}$ has complexity $\mathcal{O}(3KNlogN + 4KN)$.

Given the complexity of the operator application, each iteration of the Conjugate Gradient solver has a cost of $\mathcal{O}(3KNlogN + 4KN + 10N)$, where the $10N$ cost is representative of the remaining operations. Hence, for p maximum iterations the complexity of the Conjugate Gradient solver is $\mathcal{O}(p3KNlogN + p4KN + p10N)$.

Each iteration of Algorithm 1 requires the application of the Conjugate Gradient solver and the calculation of $(\mathbf{H}_{\mathbf{a}^{(n-1)}})^T \mathbf{y}$ with the updated version of $\mathbf{a}^{(n-1)}$, which costs $\mathcal{O}(KNlogN + 2KN)$, since the modulated and transformed values for $\mathbf{a}^{(n-1)}$ are reused. Finally $2N$ operations are needed for the recombination step.

Algorithm 1 terminates when the change its estimated solution between iterations in below a threshold, however a maximum number of iterations q can be defined.

Based on all the above, the total complexity of Algorithm 1, is $\mathcal{O}(q(3p+1)KNlogN + q(4p+2)KN + 10qpN)$ basic compute operations.

S4 Initialization for non-negative signals

In the case that the signal is known to be real, non-negative and noise free Parseval's theorem can be utilized to find an initial estimate whose energy is equal to the energy of the sought solution

$$\sum_n \sum_k y_{nk} = N \sum_n \sum_k |w_k(n) x_n|^2 = NK \sum_n^N |x_n|^2, \tag{S110}$$

where $w_k(n) \in \{1, -1, i, -i\}$ are the elements of the diagonal of $\mathbf{W}_k$.

A vector $\mathbf{r}$ with elements uniformly sampled from the interval $[0, 1]$ can be generated. The vector $\mathbf{r}$ is normalized and then multiplied with the norm of the solution vector $\mathbf{x}$, to produce the initial estimate:

$$\bar{\mathbf{a}}^0 = \bar{\mathbf{b}}^0 = \frac{\mathbf{r}}{||\mathbf{r}||} \sqrt{\frac{\sum_n^N \sum_k^K y_{nk}}{NK}}. \tag{S111}$$

This method generates a non-negative real signal with the same energy as the solution with minimal computational cost.

S5 Comparison of proposed method with PhaseSplit

The method proposed in this paper shares the same formulation as the PhaseSplit algorithm [4]. We offer a comparison of the update equations of the two methods to highlight their differences. Using the notation presented in [4] and expanding the multiplier terms, the updates of PhaseSplit are

$$\mathbf{u}^{n+1} = \mathbf{C}_{\mathbf{v}^n}^{-1}(2\lambda \mathbf{v}^n + \sum_i^m (\mathbf{h}_i^T \mathbf{v}^n) y_i \mathbf{h}_i - \lambda \mathbf{u}^n) \tag{S112}$$

and

$$\mathbf{v}^{n+1} = \mathbf{C}_{\mathbf{u}^{n+1}}^{-1}(\lambda \mathbf{u}^{n+1} + \sum_i^m (\mathbf{h}_i^T \mathbf{u}^{n+1}) y_i \mathbf{h}_i + \lambda \mathbf{u}^n - \lambda \mathbf{v}^n). \tag{S113}$$

The objective function used to derive the proposed method corresponds to $\lambda = 0$. Beyond this, the update equations of the variables are diffent because of the introduction of the recombination step. To highlight this, using the same notation as [4], we rewrite the proposed method update equations as

$$\bar{\mathbf{u}}^{n+1} = \mathbf{C}_{\mathbf{v}^n}^{-1}(\sum_i^m (\mathbf{h}_i^T \mathbf{v}^n) y_i \mathbf{h}_i) \tag{S114}$$

and

$$\mathbf{u}^{n+1} = \mathbf{v}^{n+1} = 0.5\bar{\mathbf{u}}^{n+1} + 0.5\mathbf{v}^n, \tag{S115}$$

where $\mathbf{v}$ and $\mathbf{u}$ are interchangeable.

Beyond the theoretical analysis presented above, we briefly outline the key outcomes of the experimental comparison of the proposed method and the PhaseSplit algorithm. The results presented in the experiments section of the main text correspond to the best possible case of PhaseSplit we could achieve.

In the case of noisy measurements for image size 256x256 and 512x512, both methods can attain solutions of the same quality which can be higher to the one of the SGD based methods in the noisy cases. For image size 256x256 this was attained for a choice of $\lambda = 1.8 \times 10^{11}$. However, PhaseSplit is sensitive to the choice of the parameter λ, which has to be fine tuned in order to achieve the best possible results. For example, the convergence success rates experiments of table 8 where conducted with λ set to 10^6. Returning to the case of 512x512 images, experiments with lower values for λ such as 10^{10}, still converge but slower and to a higher error. The same is true for larger values such as 10^{12}, where the convergence becomes slower and final error is also higher. This shows that there is a narrow range of λ for which PhaseSplit converges to a good solution. The presence of a different noise level will also result in different convergence behaviour for the same image size, number of masks, initialization and λ. Figure 4 in section 3, shows the convergence of the algorithm for the noiseless case for $\lambda = 1.8 \times 10^{11}$.

In comparison, the proposed method does not involve any regularization parameters and relies instead on an implicit regularization. For this reason, its convergence properties vary substantially from the ones of PhaseSplit. Finally, compared to the cases where PhaseSplit performed best, the proposed method reached the same level of accuracy, about 3 times faster.

S6 Geometric interpretation of proposed algorithm

S6.1 Alternating optimization without recombination of a and b

We first examine the case where the recombination step of the algorithm is skipped. Let $\mathbf{d}$ and $\mathbf{e}$ be the error vectors after each step of the Algorithm (we omit the superindices for clarity of notation), such as

$$\mathbf{a} = \mathbf{x} + \mathbf{d} \text{ and } \mathbf{b} = \mathbf{x} + \mathbf{e}. \tag{S116}$$

Table S1, contains key values representing the geometry of vectors $\mathbf{a}$, $\mathbf{b}$, $\mathbf{d}$ and $\mathbf{e}$ at the initial point and iterations 10, 50, 100, 500, 1000 and 2000.

Iteration	Initialization	10	50	100	500	1000	2000
$\|\|\mathbf{a}\|\|/\|\|\mathbf{x}\|\|$	1	1.2698	1.2451	1.2399	1.2343	1.2334	1.2328
$\|\|\mathbf{b}\|\|/\|\|\mathbf{x}\|\|$	1	0.8398	0.8191	0.8178	0.8131	0.8125	0.8121
$cos\theta_{\mathbf{x}}^{\mathbf{a}}$	0.8011	0.9811	0.9934	0.9960	0.9989	0.9994	0.9996
$cos\theta_{\mathbf{x}}^{\mathbf{b}}$	0.8011	0.9820	0.9935	0.9961	0.9989	0.9994	0.9996
$\|\|\mathbf{d}\|\|/\|\|\mathbf{x}\|\|$	0.6307	0.3475	0.2763	0.2593	0.2399	0.2365	0.2345
$\|\|\mathbf{e}\|\|/\|\|\mathbf{x}\|\|$	0.6307	0.2363	0.2057	0.1988	0.1913	0.1900	0.1892

Table S1: Example of evolution of variables after alternatingly estimating $\mathbf{a}$ and $\mathbf{b}$.

It is true that $\frac{||\mathbf{a}||}{||\mathbf{x}||} \geq 1$ and $\frac{||\mathbf{b}||}{||\mathbf{x}||} \leq 1$ after every iteration of the algorithm, but neither of the two sequences, $\frac{||\mathbf{a}^n||}{||\mathbf{x}||}$ and $\frac{||\mathbf{b}^n||}{||\mathbf{x}||}$, converges to 1.

Examining the angles of the vectors, we observe that $cos\theta_{\mathbf{x}}^{\mathbf{a}} \to 1$ and $cos\theta_{\mathbf{x}}^{\mathbf{b}} \to 1$ as the number of iterations increases, which means $\mathbf{a}$ and $\mathbf{b}$ tend to become aligned with the solution $\mathbf{x}$.

These facts suggest that if we set $\mathbf{a}$ and $\mathbf{b}$ to a point that geometrically lies between them (for example their mean), then the algorithm could escape from the local minimum and continue moving towards the true solution.

S6.2 Alternating optimization with recombination of a and b

The results of subsection S6.1 show that the vectors $\mathbf{a}$ and $\mathbf{b}$ could potentially be recombined to produce better estimates. The averaging recombination step is introduced and the algorithm is executed again. Table S2 provides empirical numerical results on the geometry of the algorithm.

Iteration	Initialization	1	2	3	4	5	6
$\|\|\mathbf{a}\|\|/\|\|\mathbf{x}\|\|$	1	1.3391	0.9636	0.9957	0.9999	1.0000	1.0000
$\|\|\mathbf{b}\|\|/\|\|\mathbf{x}\|\|$	1	0.8731	1.0557	1.0043	1.0000	1.0000	1.0000
$cos\theta_{\mathbf{x}}^{\mathbf{a}}$	0.8011	0.9433	0.9945	0.9999	1.0000	1.0000	1.0000
$cos\theta_{\mathbf{x}}^{\mathbf{b}}$	0.8011	0.9602	0.9947	0.9998	1.0000	1.0000	1.0000
$\|\|\mathbf{d}\|\|/\|\|\mathbf{x}\|\|$	0.6307	0.5165	0.1008	0.0080	5.6483e-5	1.1316e-8	2.2075e-12
$\|\|\mathbf{e}\|\|/\|\|\mathbf{x}\|\|$	0.6307	0.2923	0.1192	0.0081	5.6489e-5	1.1316e-8	2.2075e-12
$cos\theta_{\mathbf{e}}^{\mathbf{d}}$	1	-0.9397	-0.9943	-0.9999	-1.0000	-1.0000	-1.0000

Table S2: Example of evolution of variables at each iteration of the algorithm. The values shown are before averaging the vectors.

As in the case where the recombination step is not included, the value $||\mathbf{x}||$ is between $||\mathbf{a}||$ and $||\mathbf{b}||$ (in any order) at each iteration, and $cos\theta_{\mathbf{x}}^{\mathbf{a}} \to 1$ and $cos\theta_{\mathbf{x}}^{\mathbf{b}} \to 1$. Introducing the recombination step results in the algorithm converging to the correct solution in 6 iterations.

In the first few iterations, the error vectors $\mathbf{d}$ and $\mathbf{e}$ have comparable lengths and the cosine of their angle is close to -1. As the iterations progress and the estimates approach the solution, the vectors $\mathbf{d}$ and $\mathbf{e}$ have almost exactly the same lengths and become collinear, pointing at opposite directions.

Therefore, the vector $\frac{\mathbf{d}+\mathbf{e}}{2}$ is expected to be closer to the origin than $\mathbf{d}$ or $\mathbf{e}$. In consequence, since $\frac{\mathbf{a}+\mathbf{b}}{2} = \mathbf{x} + \frac{\mathbf{d}+\mathbf{e}}{2}$, the vector $\frac{\mathbf{a}+\mathbf{b}}{2}$ will be closer to the solution $\mathbf{x}$ than $\mathbf{a}$ or $\mathbf{b}$.

Figure S1, shows the values of the objective function, as well as the norms of the error vectors $\mathbf{d}$ and $\mathbf{e}$ at each iteration. Each iteration consists of three points: the two estimations of $\mathbf{a}$ and $\mathbf{b}$, where the value of the objective function decreases and a third one corresponding to the averaging update. Despite the objective function slightly increasing after taking the mean of $\mathbf{a}$ and $\mathbf{b}$, the values of $\|\mathbf{d}\|$ and $\|\mathbf{e}\|$ decrease.

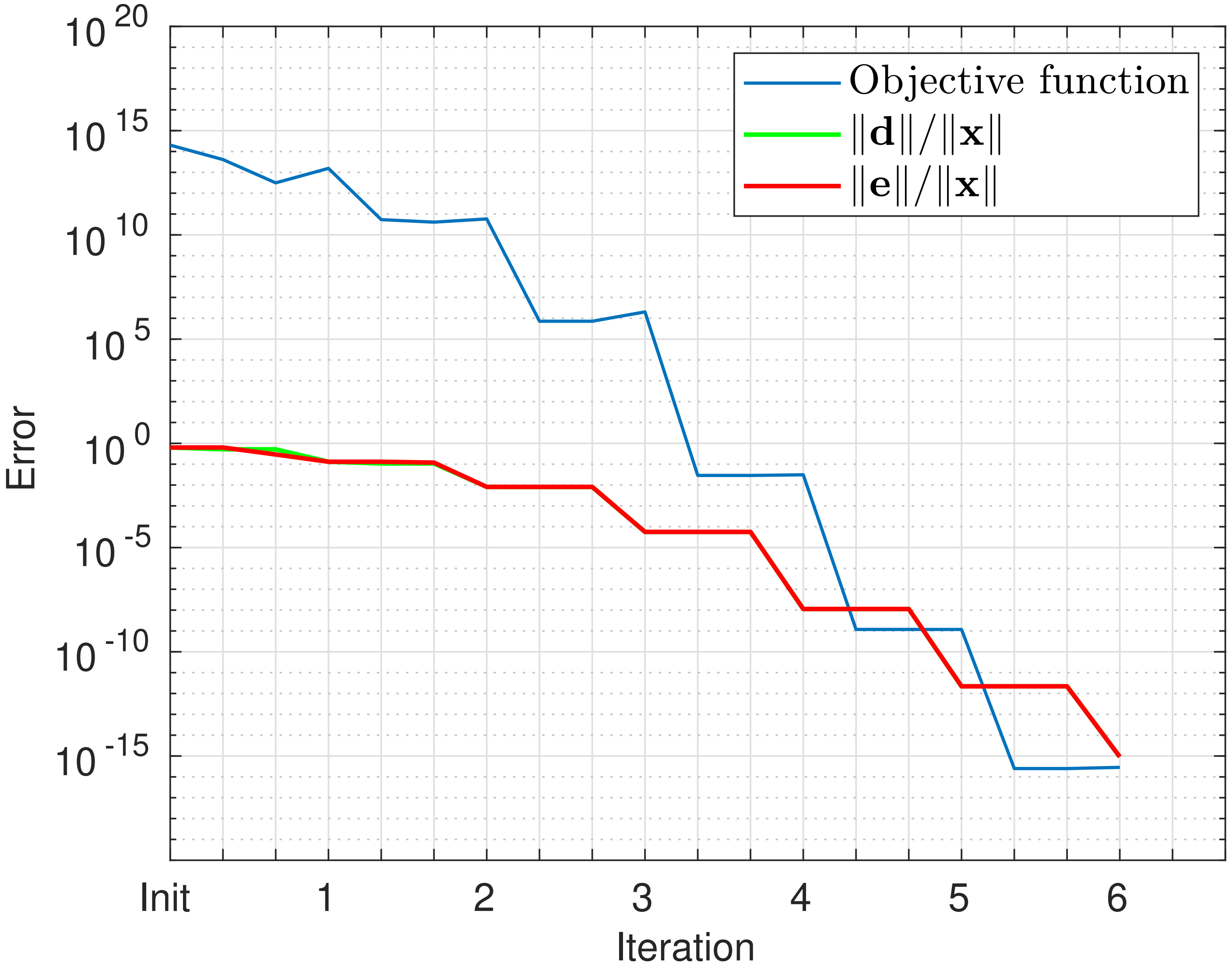

Figure S1: Values of objective function and normalized norms of vectors $\mathbf{d}$ and $\mathbf{e}$ after each step of the algorithm. The error norm decreases faster as the algorithm progresses.

References

[1] Emmanuel J. Candès, Xiaodong Li, and Mahdi Soltanolkotabi, "Phase retrieval from coded diffraction patterns," *Applied and Computational Harmonic Analysis*, vol. 39, 10 2013.

[2] E. J. Candès, X. Li, and M. Soltanolkotabi, "Phase retrieval via wirtinger flow: Theory and algorithms," *IEEE Trans. Information Theory*, vol. 61, no. 4, pp. 1985–2007, 2015.

[3] Richard Barrett, Michael Berry, Tony Chan, June Donato, Jack Dongarra, Victor Eijkhout, Roldan Pozo, Chris Romine, and Henk Van der Vorst, "Templates for the solution of linear systems: Building blocks for iterative methods," *Mathematics of Computation*, vol. 64, 09 1996.

[4] Subhadip Mukherjee, Suprosanna Shit, and Chandra Sekhar Seelamantula, "Phasesplit: A variable splitting framework for phase retrieval," in *2018 IEEE International Conference on Acoustics, Speech and Signal Processing (ICASSP)*, 2018, pp. 4709–4713.
